# New records on the rich loriciferan fauna of Trezen ar Skoden (Roscoff, France): Description of two new species of *Nanaloricus* and the new genus *Scutiloricus*

**DOI:** 10.1371/journal.pone.0250403

**Published:** 2021-05-05

**Authors:** Ricardo Cardoso Neves, Reinhardt Møbjerg Kristensen, Nadja Møbjerg

**Affiliations:** 1 Department of Biology, August Krogh Building, University of Copenhagen, Copenhagen Ø, Denmark; 2 Natural History Museum of Denmark, University of Copenhagen, Copenhagen Ø, Denmark; CIIMAR Interdisciplinary Centre of Marine and Environmental Research of the University of Porto, PORTUGAL

## Abstract

Loricifera is a phylum of microscopic animals that inhabit marine environments worldwide. Named after their conspicuous and protective lorica, the phylum was first described from Roscoff (France) in 1983 and, hitherto, it contains only 40 species. Based on data collected from Roscoff during the past four decades, we here describe two new species of *Nanaloricus*, namely *Nanaloricus valdemari* sp. nov. and *Nanaloricus mathildeae* sp. nov., as well as a new genus and species, *Scutiloricus hugoi* gen. et sp. nov. Adults of *N*. *valdemari* sp. nov. are distinguished by a pair of unique cuticular ridges, here referred to as longitudinal stripes, spanning laterally along the anterior two thirds of the dorsal lorical plate. *N*. *mathildeae* sp. nov. is characterized by strong sexual dimorphism. Specifically, the branches composing the multiform male clavoscalids are much broader as compared to other *Nanaloricus* species. The two new *Nanaloricus* species are both characterized by unique sensory organs associated with the double trichoscalids. The size and exact position of these organs differ between the two species. Adults of *Scutiloricus hugoi* gen. et sp. nov. are characterized by, among other features, a square lorica composed of six cuticular plates with a total of 14 anterior spikes, of which 12 have transverse cuticular ridges and thus appear fenestrated; laterodorsal flosculi arranged linearly; a posterior lorical region characterized by an anal field with a small anal cone flanked by a pair of spurs. Notably, mature females are characterized by a pair of seminal receptacles, a character not previously reported in Loricifera. We discuss the new findings and compare *N*. *valdemari* sp. nov. and *N*. *mathildeae* sp.nov. with other species assigned to genus *Nanaloricus*. The distinguishing features of *Scutiloricus hugoi* gen. et sp. nov. are discussed from a comparative perspective with the other genera of family Nanaloricidae.

## Introduction

The marine tidal and offshore localities near Roscoff (Brittany, France) are renowned for their biodiversity and exceptional animal life. This is exemplified by the description of and current research on the Roscoff worm, *Symsagittifera roscoffensis* (Graff, 1891) (see e.g. [1, 2]) and by investigations into the diversity and adaptations of tardigrades [3–7]. Notably, the phylum Loricifera was described in 1983 from the study of the first representative specimens found off the coast of Roscoff [8]. From this pioneering investigation a single species was described, *Nanaloricus mysticus* Kristensen, 1983, which was accommodated in the first loriciferan family to be proposed, Nanaloricidae Kristensen, 1983. Since then, 40 loriciferan species have been described from several locations worldwide and accommodated in this family or in one of the two other families described later, Pliciloricidae Higgins and Kristensen, 1986 and Urnaloricidae Heiner and Kristensen, 2009 [9–14].

Loriciferans are meiobenthic animals of microscopic size (80–800 μm in length) found in sediments from shallow-water coarse sands to deep-sea muddy bottoms at a wide range of depths [8, 11, 12, 15, 16]. Our knowledge on the diversity of loriciferan fauna is still improving as new species and life cycle stages are recurrently discovered (see e.g., [17–20]). For example, the presence of the first hermaphroditic loriciferan was only recently confirmed [21]. All loriciferans found so far are free living forms.

The loriciferan life cycle is complex and involves larval and postlarval stages, which succeed through development into adult forms [12, 22]. Members of the family Nanaloricidae possess the simplest life cycle. Briefly, the embryo develops into a so-called Higgins larva that molts several times until it metamorphoses into a postlarva (= juvenile), which finally molts into a male or a female that reproduces sexually and thus completes the life cycle [12]. On the contrary, Pliciloricidae can undergo both sexual and asexual reproduction. The asexual life cycle involves several intermediate stages and reduced larval forms [17, 23, 24].

Adult loriciferans possess a body divided into a head (mouth cone and introvert), neck, thorax and abdomen [11, 12]. Several rows of spine-like scalids of various shapes and sizes are present on the introvert and neck, while the thorax is covered with a thin cuticle and lacks any appendages. The latter region is inconspicuous in Nanaloricidae. The abdominal region is encased in a lorica, which is a cuticular exoskeleton composed of 6–20 plates (Nanaloricidae) or several plicae and folds (Pliciloricidae). The newly described genus and species *Fafnirloricus polymetallicus* exhibits the highest number of lorica plates ever found in a nanaloricid, i.e. 20 [13]. Another prominent life cycle stage, mentioned above, is the Higgins larva, which has an outer morphology that resembles adults, but in addition possesses a pair of toes located at the most posterior region of the abdomen. In addition, another type of larval stage, known as the Shira larva, has been described and listed as *incertae sedis* within Loricifera because of its unique morphological features [25].

In 2013, specimens of *Nanaloricus* sp.—an undescribed species collected off Roscoff—were investigated to describe the myoanatomy of nanaloricid loriciferans [26]. This unnamed species has been known since 1985 (R.M. Kristensen, pers. obs.) and, referred to as *Nanaloricus* n. sp., it was also examined in a molecular-based study to assess the phylogenetic relationships of Loricifera [27]. The new species was never properly described. In addition, two other, yet, undescribed nanaloricid species have been known from Roscoff since 1985, when the genus *Armorloricus* was found ([28]; R.M. Kristensen, pers. obs.). Hitherto, none of these three species has been thoroughly examined, although specimens of all of them have been collected again (all authors, pers. obs.). Indeed, specimens of two of the three new nanaloricid species have been recurrently collected and observed between 1985 and 2020, while the third species is less common and appears more distantly related to other nanaloricids. Here, we describe *Nanaloricus valdemari* sp. nov., *Nanaloricus mathildeae* sp. nov., and *Scutiloricus hugoi* gen. et sp. nov., of which the former corresponds to the undescribed species previously used in both the myoanatomical and the phylogenetic studies mentioned above. Our findings on the morphology of the new species are discussed and compared with that of other nanaloricid species, including the type species *Nanaloricus mysticus*. The discovery of three new nanaloricid species in the coastal area off Roscoff substantially increases the number of verified loriciferan species present in the subtidal area that includes the type locality of Loricifera. This further substantiates the observation that marine localities off the coast of Roscoff, such as the subtidal shell dune Trezen ar Skoden, indeed represent a biodiversity hotspot for Loricifera.

## Materials and methods

### Collection of specimens and light microscopy

No specific permits were required for the described field studies, as none of the investigated species are included in any endangered list, at national or international levels. Specimens of adult *Nanaloricus valdemari* sp. nov., *Nanaloricus mathildeae* sp. nov. and *Scutiloricus hugoi* nov. et sp. nov. were obtained from very clean, coarse shell gravel collected off the coast of Roscoff, France, in a location known as Trezen ar Skoden (48°45’55”N/04°06’45”W to 48°45’30”N/04°06’25”W; see, e.g., [28]). Samples were taken between 1985 and 2020 with a Sanders dredge at 42–55 m water depths. The samples were soaked and gently stirred in fresh water, to ensure the release of loriciferans and other meiofauna from the sediment grains due to osmotic shock. Subsequently, the meiofauna was extracted by decantation through 32, 45 or 63 μm mesh nets (mermaid bras), transferred to Petri dishes with filtered sea water and the loriciferan specimens were sorted out under a stereomicroscope. A total of 15 living loriciferans, belonging to the two new *Nanaloricus* species, were transferred to glass slides in sea water and photographed under cover slips using a DP27 camera mounted on an Olympus BX53 compound microscope with differential interference contrast (DIC) optics. These specimens have been preserved in RNA later or prepared for DNA extraction for future studies (note that no molecular data are reported in the present investigation).

Loriciferan specimens were fixed in 4% paraformaldehyde (PFA) buffered either with Borax or a 0.1 mol l^-1^ phosphate buffer saline solution and mounted in glycerine, Vectashield (Vector Laboratories) or Fluoromount G (SouthernBiotech) on glass slides. Whole mount preparations were sealed with Glyceel and investigated with an Olympus BX51 microscope fitted with phase contrast (PC) and DIC optics. Photographs were taken with an Olympus DP20 Cell zoom digital camera. Alternatively, a Nikon Microphot-Fx microscope equipped with interference contrast optics was used to take photographs of internal details of specimens of *Scutiloricus hugoi* gen. et sp. nov. temporarily mounted in distilled water. The drawings were made with the aid of a drawing tube mounted on a Wild M20 microscope with phase contrast optics.

### Scanning Electron Microscopy (SEM)

After extraction and fixation in PFA, the specimens were briefly washed with distilled water and dehydrated through a graded series of alcohol and subsequently transferred to acetone through a graded ethanol/acetone series. Subsequently, specimens were critical point dried using an Autosamdri-815 dryer (Tousimis Research Corporation, Maryland, USA) or a BAL-TEC 030 dryer (Bal-Tec AG, Balzers, Liechtenstein) with carbon dioxide as intermediate. Finally, the dried specimens were mounted on aluminium stubs with sticky carbon pads and sputter-coated with platinum/palladium alloy and analysed with a JEOL JSM-840 Scanning electron microscope or a JEOL JSM-6335F Field Emission Scanning electron microscope.

All examined type material is deposited at the Zoological Museum, Natural History Museum of Denmark (NHMD), University of Copenhagen.

### Nomenclatural acts

The electronic edition of this article conforms to the requirements of the amended International Code of Zoological Nomenclature (ICZN), and hence the new names contained herein are available under that Code from the electronic edition of this article. This published work and the nomenclatural acts it contains have been registered in ZooBank, the online registration system for the ICZN. The ZooBank LSIDs (Life Science Identifiers) can be resolved and the associated information viewed through any standard web browser by appending the LSID to the prefix “http://zoobank.org/”. The LSID for this publication is: urn:lsid:zoobank.org:pub: 36176448-9BF3-4387-9BE5-15CCA575C1B1. The electronic edition of this work was published in a journal with an ISSN, and has been archived and is available from the following digital repositories: PubMed Central, LOCKSS, Marine Data Archive (MDA).

## Results

### Systematics

Phylum: Loricifera Kristensen, 1983

Order: Nanaloricida Kristensen, 1983

Family: Nanaloricidae Kristensen, 1983

Genus: *Nanaloricus* Kristensen, 1983

(Type species: *Nanaloricus mysticus* Kristensen, 1983)

***Nanaloricus valdemari*** sp. nov.

(Figs 1–7).

**Fig 1:**
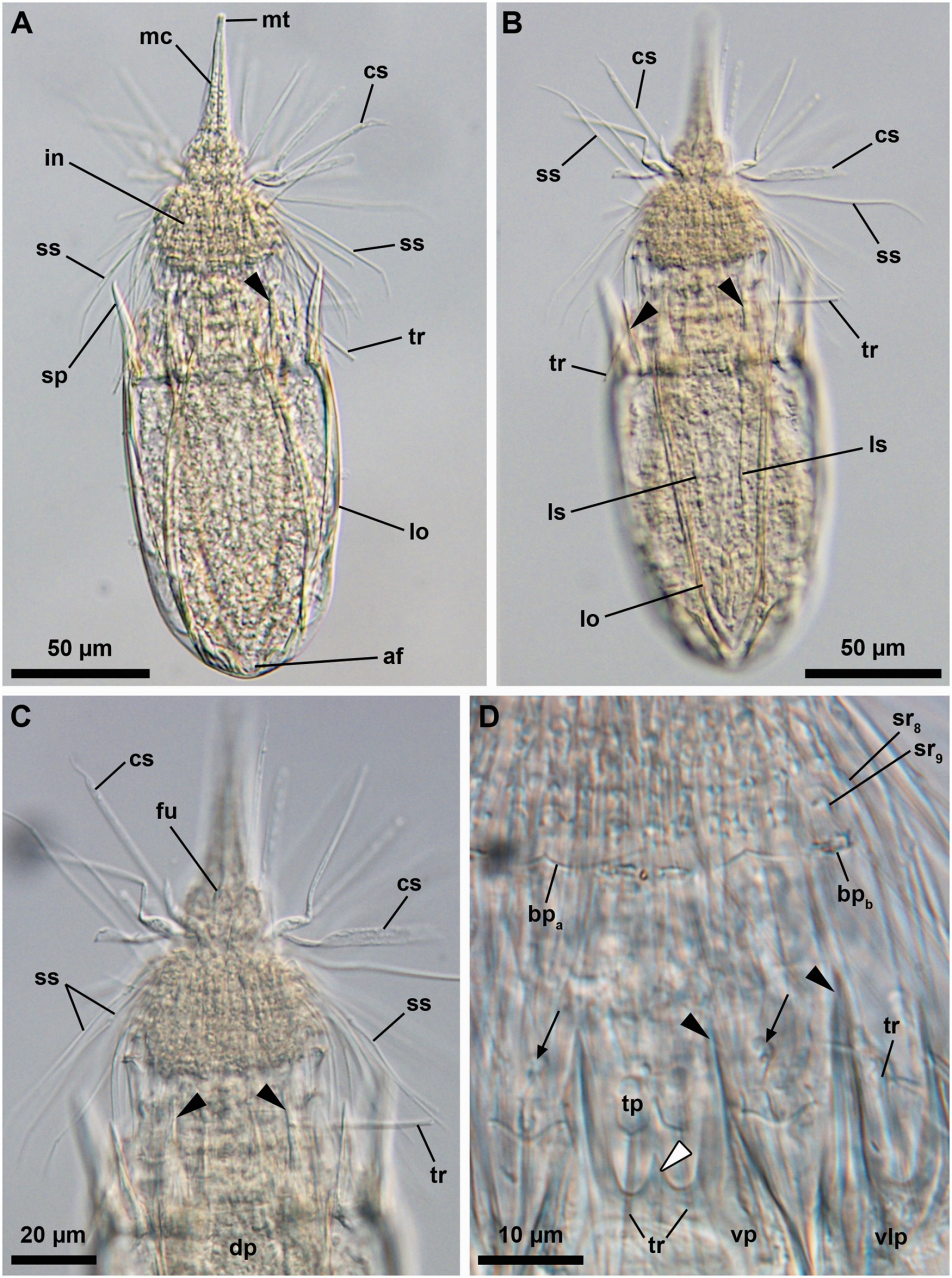
Light micrographs of the holotypic adult male of *Nanaloricus valdemari* sp. nov. Anterior faces up in all aspects. (A) Ventral view of the extended specimen. Note that the mouth tube is not fully extended. (B) Dorsal view of the specimen. Note the pair of longitudinal stripes (ls) spanning the anterior two thirds of the dorsal plate of the lorica. (C) Anterior region of the specimen, dorsal view. (D) Close-up of the posterior region of the introvert and neck, ventral view. White arrowhead points to the small, midventral anterior spike. Arrows point to trichoscalid sensory organs. Abbreviations: af, anal field; bp_a/b_, basal plate of type a or b; cs, clavoscalid; dp, dorsal plate; fu, oral furca; in, introvert; lo, lorica; mc, mouth cone; mt, mouth tube; sp (and black arrowheads), anterior spike; sr_8/9_, spinoscalid of 8^th^ or 9^th^ row; ss, spinoscalid; tp, trichoscalid plate; tr, trichoscalid; vlp, ventrolateral plate; vp, ventral plate.

**Fig 2:**
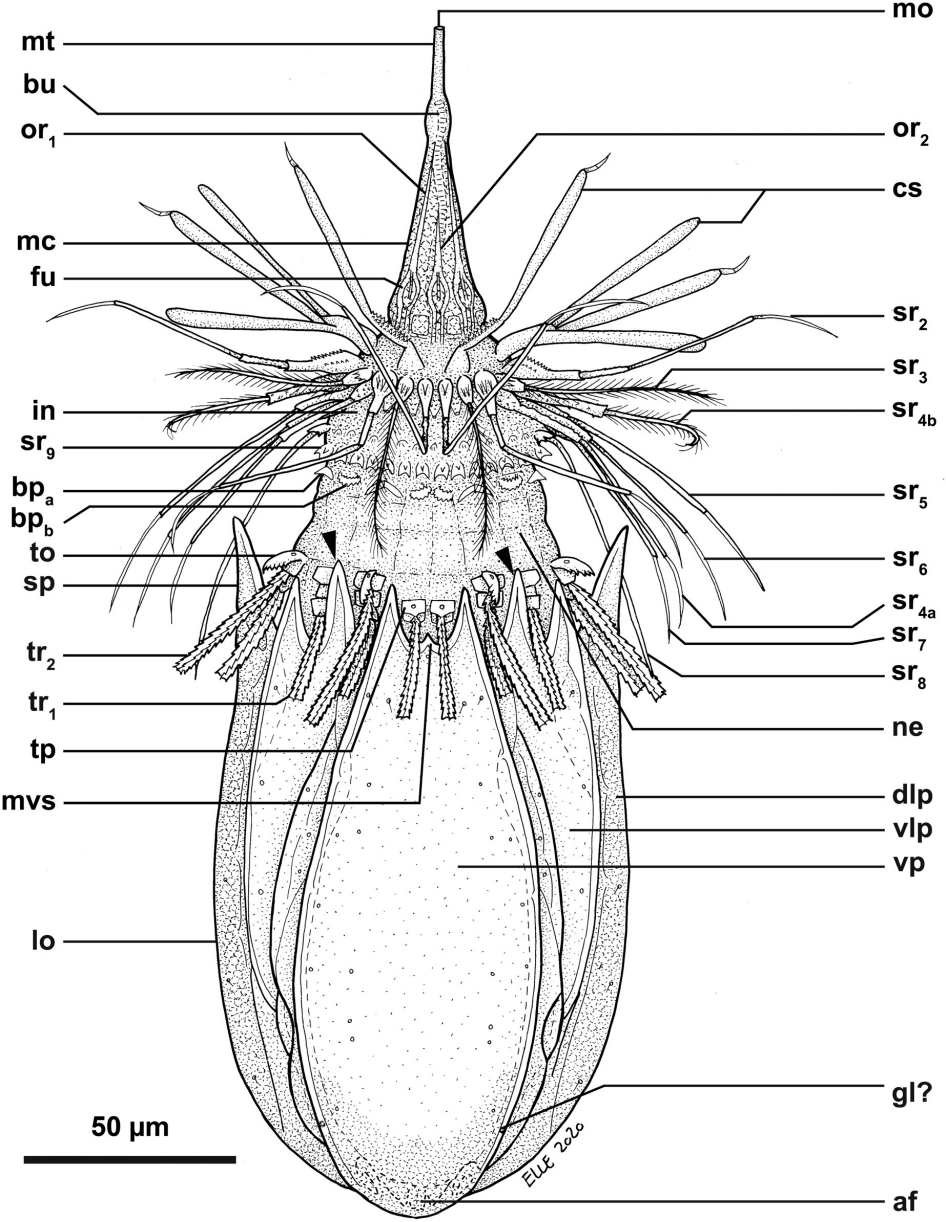
Line art drawing of the adult male habitus of *Nanaloricus valdemari* sp. nov. Ventral view of the body, anterior faces up. Note that only a selected number of scalids from rows 1 to 8 are represented for clarity. Abbreviations: af, anal field; bp_a/b_, basal plates of type a and b; bu, buccal tube; cs, clavoscalid; dlp, dorsolateral plate; fu, oral furca; gl?, putative gland outlet; in, introvert; lo, lorica; mc, mouth cone; mo, mouth aperture; mt, mouth tube; mvs, midventral anterior spike; ne, neck; or_1/2_, oral ridges of type 1 and 2; sr_2–9_, spinoscalids of 2^nd^ to 9^th^ row (including sr_4a/b_, i.e. type a and b spinoscalids of the 4^th^ row); sp (and black arrowheads), anterior spike; to, trichoscalid sensory organ; tp, trichoscalid plate; tr_1_, single trichoscalid; tr_2_, double trichoscalid; vlp, ventrolateral plate; vp, ventral plate.

**Fig 3:**
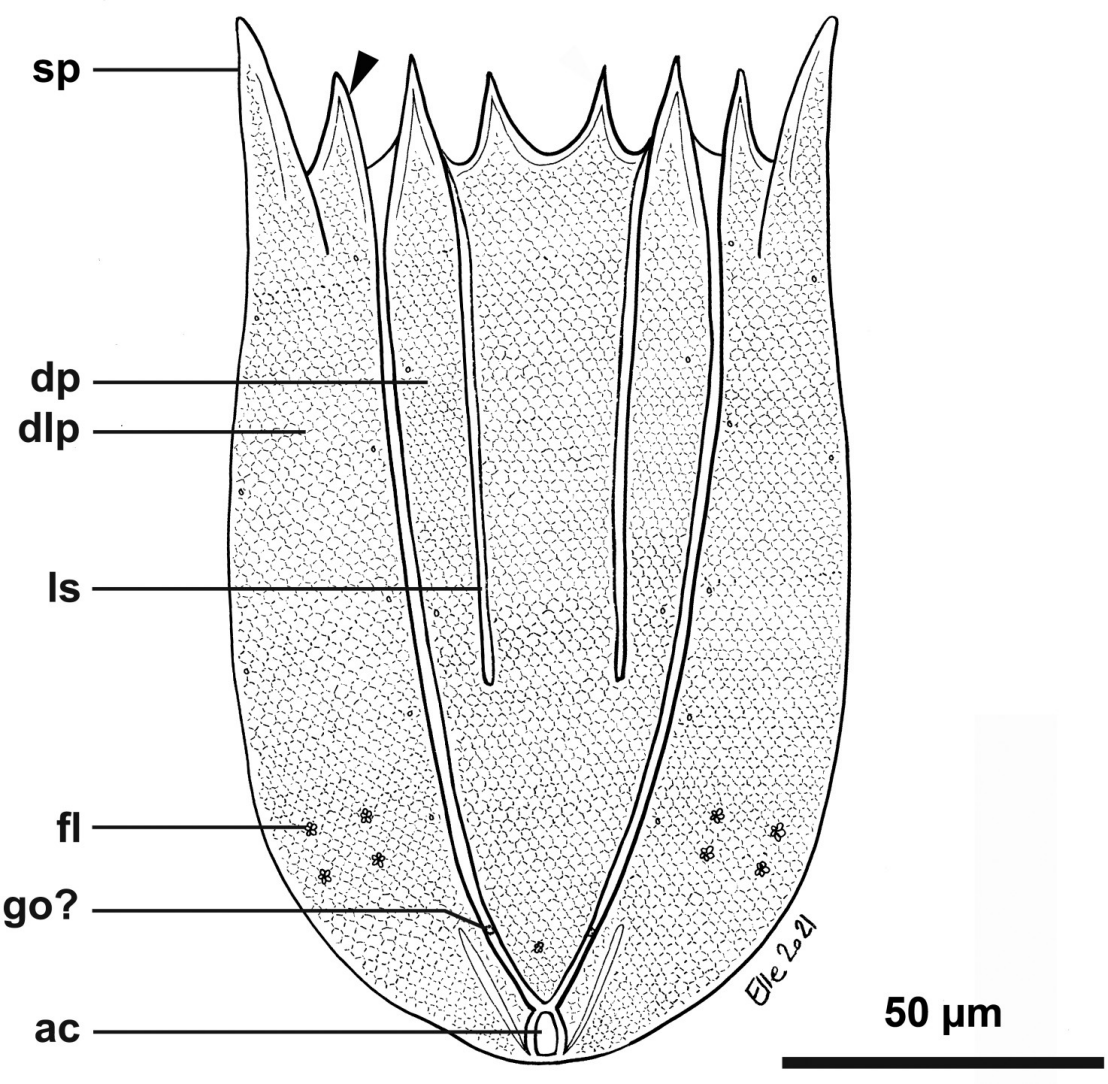
Line art drawing of the adult male partial habitus of *Nanaloricus valdemari* sp. nov. Dorsal view of the lorica, anterior faces up. Abbreviations: ac, anal cone; dlp, dorsolateral plate; dp, dorsal plate; fl, flosculum; go?, putative gonopore; ls, longitudinal stripe; sp (and arrowhead), anterior spike.

**Fig 4:**
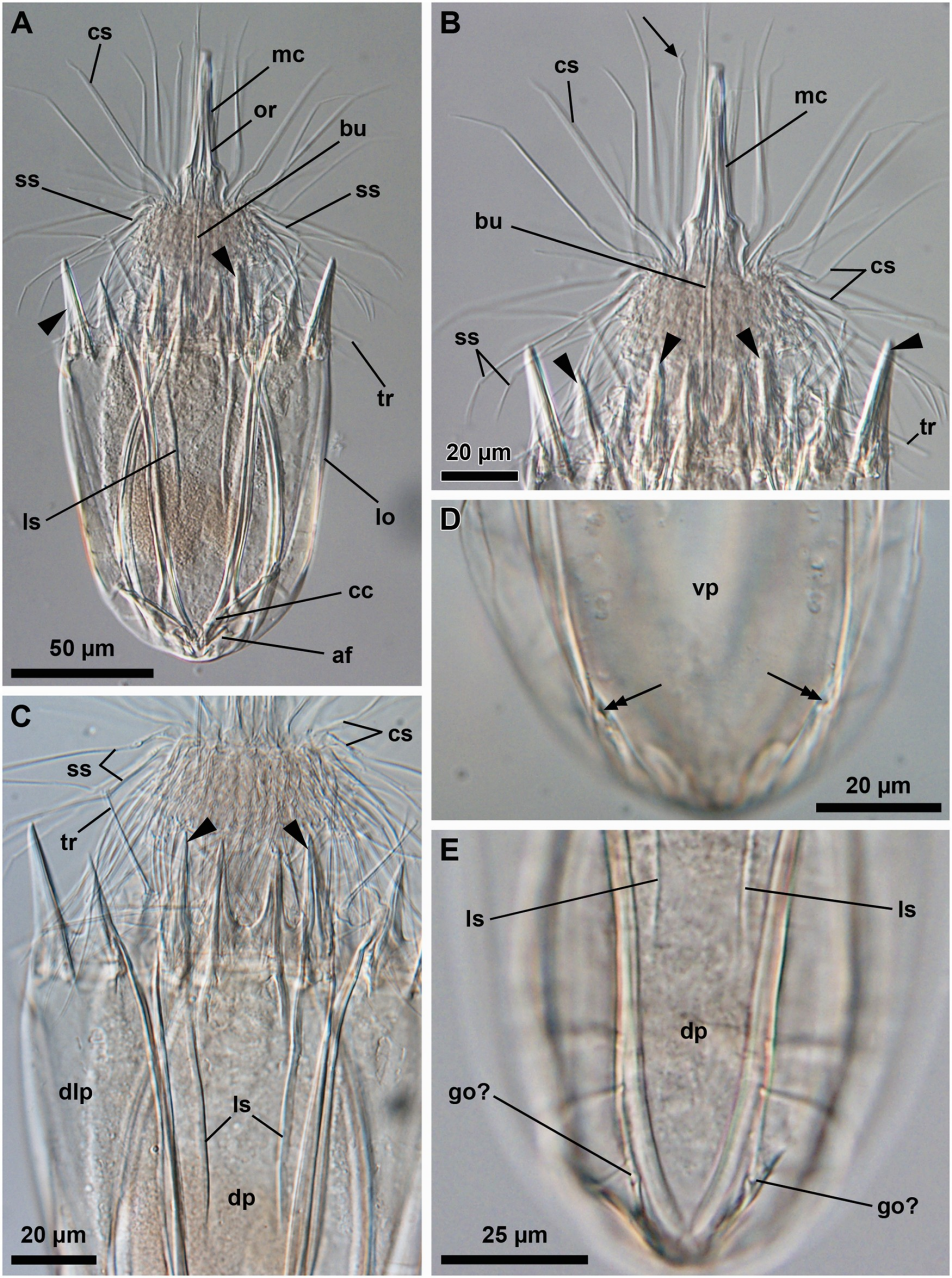
Light micrographs of the allotypic adult female of *Nanaloricus valdemari* sp. nov. Anterior faces up in all aspects. (A) Overview of the specimen, dorsal view. Note that the mouth tube is not fully extended. Note also the pair of longitudinal stripes (ls) spanning the anterior two thirds of the dorsal plate of the lorica. (B) Close-up of the anterior region of the specimen, dorsal view. Arrow points to the two most distal segments of a clavoscalid. (C) Close-up of the introvert, neck and anterior region of the abdomen, dorsal view. (D) Posterior region, ventral view. Double-headed arrows point to large pores (gland outlets?) situated on the posterior region of the ventral plate. (E) Posterior region, dorsal view. Note the putative gonopores (go?) located on the posteriormost region of the dorsolateral plates. Abbreviations: af, anal field; bu, buccal tube; cc, cuticularized crest; cs, clavoscalid; dlp, dorsolateral plate; dp, dorsal plate; lo, lorica; mc, mouth cone; or, oral ridge; sp (and black arrowheads), anterior spike; ss, spinoscalid; tr, trichoscalid; vp, ventral plate.

**Fig 5:**
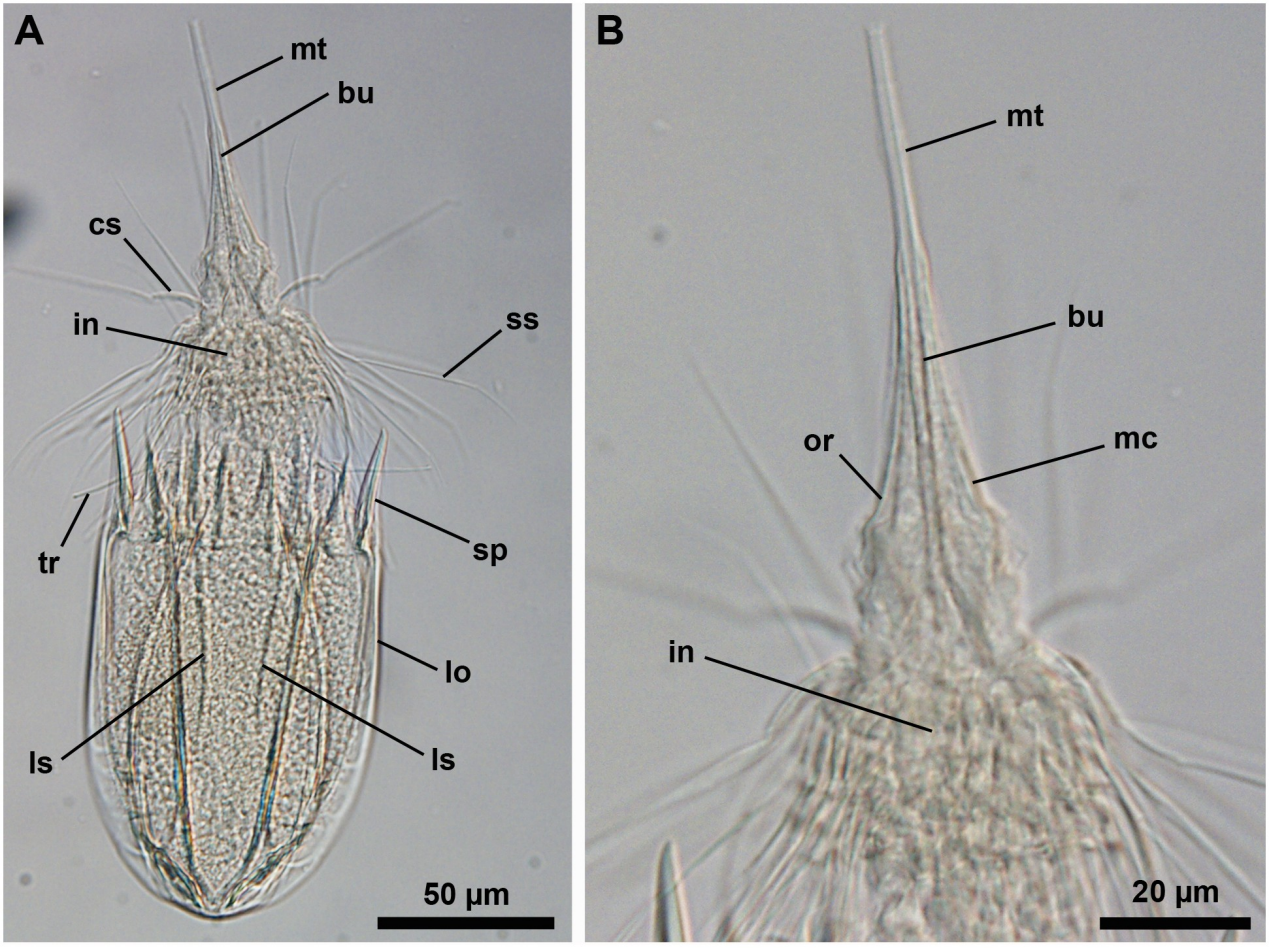
Light micrographs of a paratypic adult female of *Nanaloricus valdemari* sp. nov. Anterior faces up in both aspects, dorsal view. (A) Overview of the specimen with extended introvert and fully extended mouth tube. (B) Close-up of the anterior region of the body. Abbreviations: bu, buccal tube; cs, clavoscalids; in, introvert; lo, lorica; ls, longitudinal stripe; mc, mouth cone; mt, mouth tube; or, oral ridge; sp, anterior spike; ss spinoscalid; tr, trichoscalid.

**Fig 6:**
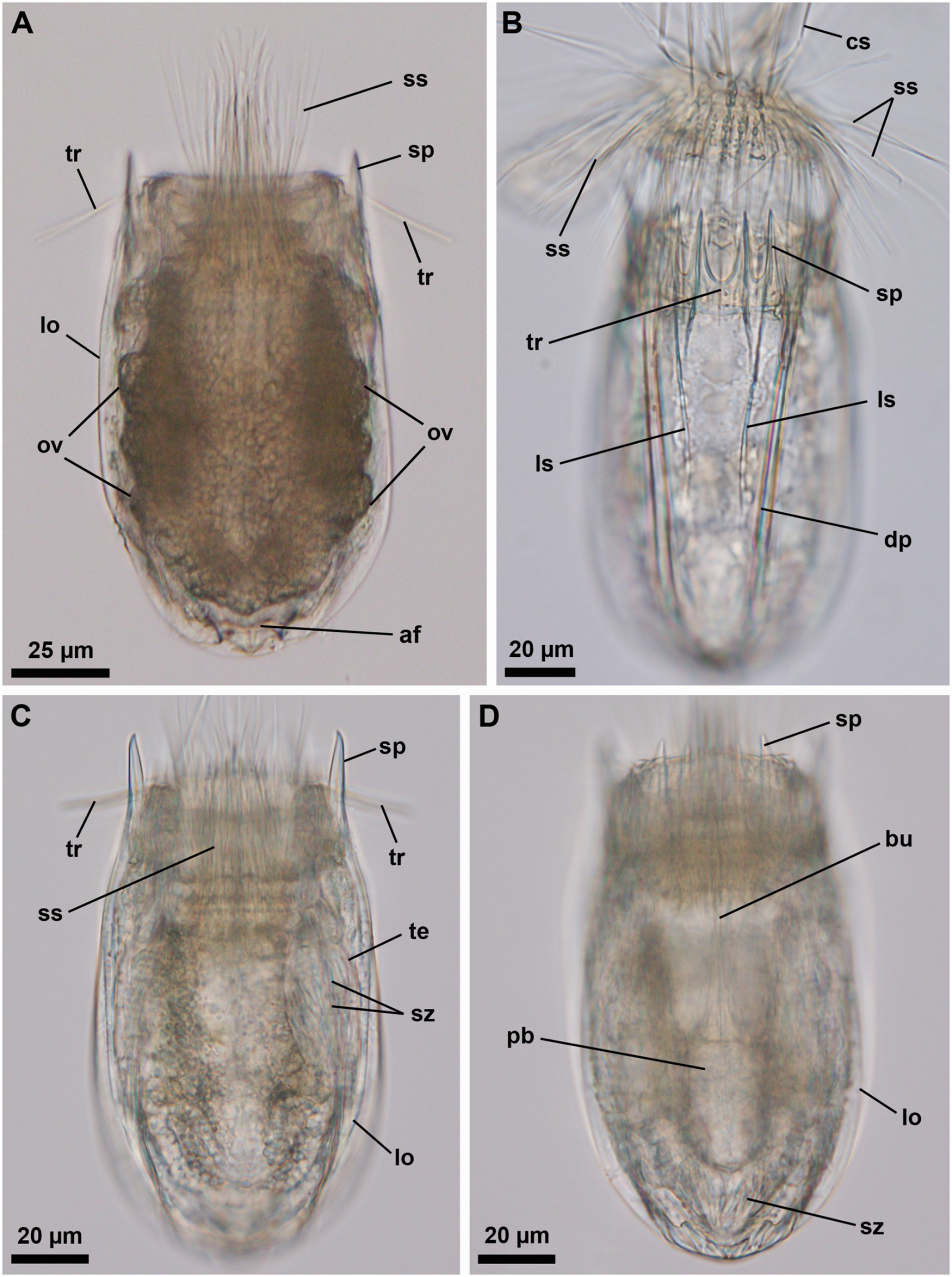
Light micrographs of three live specimens of *Nanaloricus valdemari* sp. nov. Anterior faces up in all aspects. (A) Overview of a female adult with head almost fully retracted. Internally, note the presence of the large ovaries (ov) in the abdomen. (B) Overview of a female adult (different from that shown in A). Note the pair of longitudinal stripes (ls) spanning the anterior two thirds of the dorsal plate of the lorica. (C and D) Overview of a male adult with head almost fully retracted. Note the presence, within the abdomen, of a testis (te) containing spermatozoa (sz). Note also the relative position of the pharyngeal bulb (pb) in this retracted animal. Abbreviations: af, anal field; bu, buccal tube; cs, clavoscalid; dp, dorsal plate; lo, lorica; sp, anterior spike; ss spinoscalid; tr, trichoscalid.

**Fig 7:**
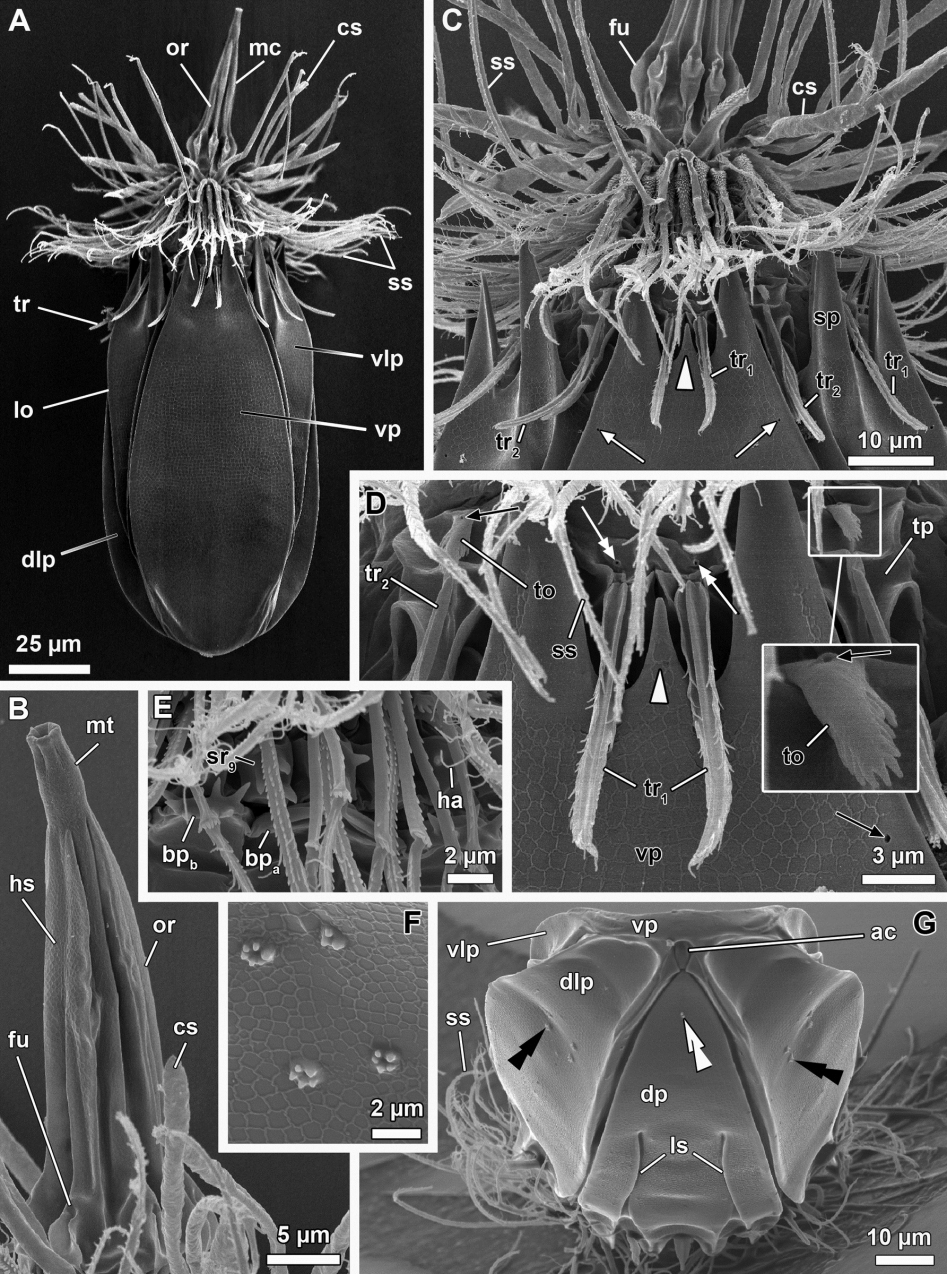
Scanning electron micrographs of three paratypic adult males of *Nanaloricus valdemari* sp. nov. (A) Overview of extended specimen, ventral view, anterior faces up. Same specimen as in C and D. Note the multiform clavoscalids: the ventralmost pair is unbranched (and similar to the eight female clavoscalids), while the others are divided into three branches. (B) Close up of the mouth tube (mt). Same specimen as in E. Note the honeycomb sculpture (hs) of the cuticle. (C) Anterior region of the body, ventral view. Note the difference between single (tr_1_) and double trichoscalids (tr_2_). White arrowhead points to the small midventral anterior spike. White arrows point to lorical pores. (D) Close up of the midventral pair of single trichoscalids, each of which protrudes from a plate characterized by a large pore (white double-headed arrow). The inset shows a magnification of the double trichoscalid sensory organ (to), which possesses a small pore (black arrows). (E) Close up of spinoscalids of 9^th^ row (sr_9_) and basal plates, one of each type (bp_a_ and bp_b_). Note the long hairs (ha) scattered along the length of the distal segments of a spinoscalid of 7^th^ (?) row. (F) Detail of a cluster of flosculi arranged in a rectangular pattern on the dorsolateral plate. Same specimen as in G. (G) Overview of the posterodorsal region of the lorica. Note the pair of longitudinal stripes (ls) that span the anterior two thirds of the dorsal plate (dp). Black double arrowheads indicate the clusters of flosculi situated on the dorsolateral plates (dlp), while the white double arrowheads point to the small flosculum situated on the dorsal plate. Abbreviations: ac, anal cone; cs, clavoscalid; dlp, dorsolateral plate; fu, oral furca; lo, lorica; mc, mouth cone; or, oral ridge; ss, spinoscalid; sp, anterior spike; tp, trichoscalid plate; tr, trichoscalid; vlp, ventrolateral plate; vp, ventral plate.

urn:lsid:zoobank.org:act: A0E2CDB1-6F62-4F86-82DF-43F2A06ED519

#### Synonymy

*Nanaloricus* sp. *sensu* [26]; *Nanaloricus* n. sp. *sensu* [27].

#### Material examined

*Holotype* (Fig 1). Adult male collected on 7 February 2013 at the type locality at ca. 45 m water depth, mounted in glycerin on a glass slide, and deposited at the Natural History Museum of Denmark under accession number NHMD-678720.

*Allotypic paratype* (Fig 4). Adult female collected on 15 May 2013 at the type locality at ca. 50 m water depth, mounted in glycerol on a glass slide, and deposited at the Natural History Museum of Denmark under accession number NHMD-678736.

*Paratypes*. 27 adults (5 males, 21 females, 1 of unkown gender) and 6 tentatively assigned postlarvae collected at the type locality between 12 July 1985 and 15 May 2013 at 43–55 m water depths. The 33 paratypic specimens are mounted in glycerin, Vectashield or Fluoromount-G on glass slides, and deposited at the Natural History Museum of Denmark under the access numbers NHMD-677708 to NHMD-677713, NHMD-677716 to NHMD-677719, NHMD-677721 to NHMD-677734 and NHMD-677737 to NHMD-677744. The specimen registered with access number NHMD-677725 is shown in Fig 5. In addition, three adult males collected at the type locality on 12 July 1985 at ca. 55 m water depth, and mounted on SEM stubs (NHMD-866002 to NHMD-866004) were analyzed for comparative purposes (Fig 7).

*Additional reference material* (Fig 6). In addition to the fixed material examined, 11 non-type specimens (one tentatively assigned postlarva, four males and six females), collected at the type locality in April 2019 and August 2020, were observed and photographed alive.

*Habitat and distribution*. Marine sediments composed of clean shell gravel at type locality.

*Type locality*. Trezen ar Skoden, Roscoff, France, (48°45’55”N, 04°06’45”E).

*Etymology*. The species is named after Valdemar Møbjerg Boslev Kristensen, who is grandson and nephew to the middle author and last author, respectively.

The following description will solely focus on adult specimens, as postlarvae have only been tentatively assigned to species (see section below: *"Notes on the postlarvae found at Trezen ar Skoden"*).

#### Diagnosis

*Adults*. (1) mouth cone with 8 oral ridges of different length and characterized by posterior sclerotized oral furcae, a telescopic mouth tube, and a well-defined honeycomb sculpture; (2) introvert with 9 rows of scalids; (3) first row with eight clavoscalids that differ between males (multiform, broad or slender, all branched except for the midventral pair) and females (four-segmented, slender, unbranched); (4) second row with 9 four-segmented, leg-like spinoscalids; (5) third row with 7 two-segmented, feather-like scalids; (6) fourth row with 16 spinoscalids of two types: 8 two-segmented, leg-like scalids (type A) alternate with 8 two-segmented spinoscalids with feather-like distal segment (type B); (7) fifth to seventh rows all similar, each row with 30 leg-like, three-segmented scalids; (8) eighth row with 30 very long unsegmented spinoscalids with a small bulbous base; (9) ninth row with 30 small, teeth-like scalids characterized by three cuspid-like protrusions; (10) neck with 8 single trichoscalids alternating with 7 double trichoscalids, with both the single and each of the double trichoscalids protruding from a single, trapezoid trichoscalid plate; (11) trichoscalid plates of the upper appendages of ventrally situated trichoscalids characterized by a short sensory organ with serrated margins and a small, anteroproximal pore; (12) lorica composed of six cuticular plates, with honeycomb sculpturing and bearing 14 large anterior spikes and a small midventral spike; (13) dorsal plate characterized by two narrow, longitudinal stripes spanning laterally along its anterior two thirds; (14) 9 flosculi (4 laterodorsal pairs and 1 dorsal) present posteriorly on the dorsal side of the lorica (dorsolateral and dorsal plates); (15) posterior region of the lorica characterized by a small anal cone, a pair of putative gonopores located postero-dorsally and a pair of ventrally located pores (gland outlets?).

#### Description

*Body (*Figs 1–7*)*. Divided into head (mouth cone and introvert), neck, thorax, and abdomen. The holotypic adult male (Fig 1) is 235 μm long, including the mouth cone, and 74 μm wide.

*Mouth cone (mc*, Figs 1, 2, 4, 5 *and* 7*)*. Long (ca. 60 μm in length), and with three distinct sections. The proximal section is broad, short and with eight conspicuous, sclerotized oral furcae (fu) arranged radially (Figs 2 and 7C). These eight furcae are of identical structure and they each extend into an oral ridge (Fig 7A–7C). The middle section is long, conical and characterised by the eight oral ridges of different lengths (Fig 2). Four primary oral ridges (or_1_) are long and span the whole middle section, while four secondary oral ridges (or_2_) are short and span only the posterior three quarters of the middle section (Figs 2, 4 and 7A and 7B). Both the proximal and middle sections are characterized by a well-defined honeycomb sculpture of the cuticle (Figs 2 and 7B). The distal section consists of a seemingly short mouth tube (mt), which is slightly bulbous posteriorly and ends with a terminal mouth aperture. However, the mouth tube is telescopic and can extend outside of the mouth cone, significantly increasing its total length (Fig 5). There are no oral stylets.

**Fig 8:**
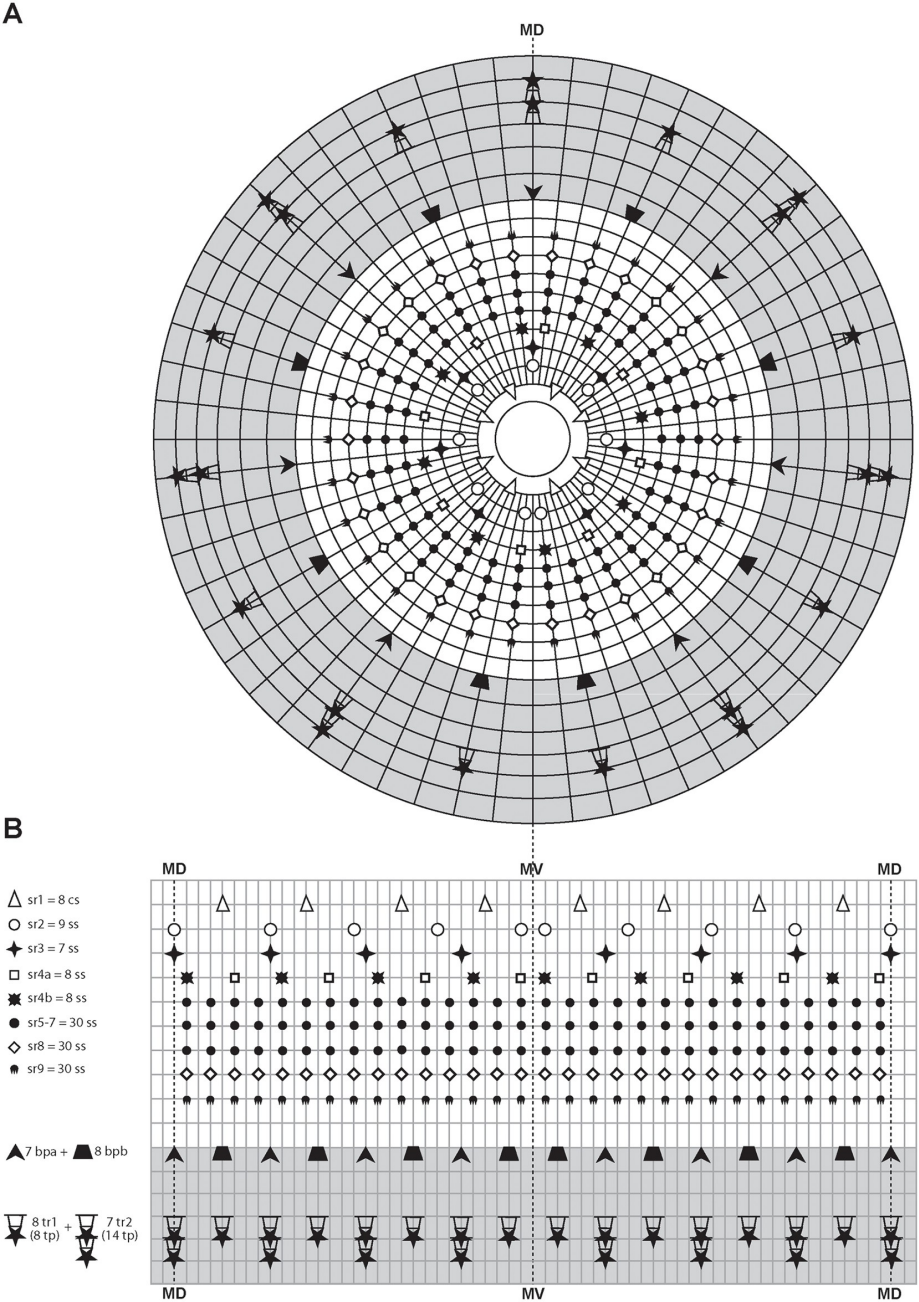
Schematic diagrams of the distribution of introvert and neck appendages in adult forms of *Nanaloricus valdemari* sp. nov. and *Nanaloricus mathildeae* sp. nov. Note that for sake of clarity clavoscalid sexual dimorphism is not depicted. Note also that the shaded area indicates the neck region. (A) Polar diagram. (B) Planar projection. Abbreviations: bp_a/b_, basal plates of type a or b; cs, clavoscalid; MD, middorsal line; MV, midventral line; sr_1–9_, scalids of 1^st^ to 9^th^ row; ss, spinoscalid; tp, trichoscalid plate; tr_1_, single trichoscalid; tr_2_, double trichoscalid.

*Introvert (in*, Figs 1, 2, 4, 5 *and* 7*)*. Round in shape and characterized by nine rows of scalids arranged radially (Figs 1, 2, 4 and 5–8).

First row (sr1, Fig 8) with eight clavoscalids (cs) that differ between males and females. In females (Figs 4 and 5), the clavoscalids are divided into four segments. The first, most proximal segment is relatively short and consists of a conical base that protrudes from the introvert and extends distally into a curved, cylindrical shape with many small papillae arranged anteriorly. The second segment, which is the longest segment of the clavoscalid, is club-shaped and slightly serrated anteriorly. The third segment is very short and thin (Figs 2 and 4B). The fourth segment is also very short and ends as a spinose tip (Figs 2 and 4B). In males (Figs 1–3 and 7) the clavoscalids are multiform. The most ventral pair is exactly as those of the female. The other six clavoscalids possess a robust base and are divided into primary, secondary and tertiary branches. Both the secondary and tertiary branches are broad and flat. However, the former has a thin base and is slightly broader than the latter. The primary branch is three-segmented and very similar to the three most distal segments of the ventral pair of clavoscalids. Indeed, the primary branch possesses distally two short, slightly curved segments: a thin one followed by a terminal spine-like segment.

The second row (sr_2_, Fig 8) consists of nine leg-like spinoscalids (ss) divided into four segments (Fig 2). The first, most proximal segment has a semiround base with a row of papillae arranged laterally on the most proximal region. The base narrows distally to form a cylindrical, short region. The second segment is short and cylindrical with many small papillae. The third segment is slightly thinner and long, representing approximately one half of the spinoscalid. The most distal segment, which represents one quarter of the spinoscalid, is slightly curved and terminates as a spinose tip. Noteworthy, the two midventral and the two ventrolateral spinoscalids of the second row are thinner than all other spinoscalids of the same row. Their bases are characterized by 1–3 small spikes.

The third row (sr_3_, Fig 8) is composed of seven short, two-segmented feather-like spinoscalids (Fig 2). The proximal segment is a small, conical base with several large papillae (up to eight?) arranged anteriorly. The distal segment is relatively long and possesses numerous short, curved hairs.

The fourth row (sr_4_, Fig 8) consists of 16 spinoscalids of two types: 8 type A leg-like spinoscalids (sr_4a_) alternating with 8 type B feather-like spinoscalids (sr_4b_) with numerous short, curved hairs (Fig 2). The type A spinoscalids are divided into three segments. The first, most proximal segment has a short, round base with several small papillae distributed in a scattered manner. The base narrows distally into a cylindrical, short region. The second segment is thin, serrated posteriorly and rather long, representing half of the total length of the spinoscalid. The third, most distal segment terminates as a thin tip. The type B spinoscalids are two-segmented, each composed of a proximal and a distal segment of similar length. The proximal segment has a round base with several densely distributed papillae. This segment narrows distally to form a cylindrical region with serrated margins. The distal segment has numerous short hairs and, hence, appears feather-like. It terminates in a hook-shaped tip with two short hairs. The fifth to seventh rows (sr_5-7_, Fig 8) are each composed of 30 three-segmented, leg-like spinoscalids (Fig 2). The most proximal segment has a short, narrow base. The middle segment is thin and very long, representing more than half of the total length of the scalid. The most distal segment is relatively long, thin and terminates as a spine-like tip. The two most distal segments have finely serrated margins and several long hairs (ha, Fig 7E) scattered along their length. The hairs are only seen by SEM.

The eighth row (sr_8_, Fig 8) consists of 30 unsegmented whipe-like spinoscalids with a small bulbous base with a few papillae (Fig 2). These spinoscalids have finely serrated margins and several long hairs scattered along their length (only seen by SEM), and they terminate as a thin tip.

The ninth row (sr_9_, Fig 8) consists of 30 small, teeth-like scalids (well discernible by SEM, Figs 2 and 7E). Each of these scalids possesses an oval anterior edge and three cuspid-like projections that protrude laterally and extend backwards from the posterior edge.

*Neck (ne*, Figs 2 *and* 8*)*. Composed of at least four rows of neck flat plates, which are divided into columns by several longitudinal folds (Fig 2). A row of 15 small basal plates is present in the most anterior region of the neck, immediately under the ninth row of spinoscalids (Figs 1D and 7E). This row consists of seven triangular plates (bp_a_) alternating with eight oval plates (bp_b_), each with two rows of teeth arranged transversely. The two midventral basal plates are of the latter type (i.e., bp_b_). Each basal plate is arranged in the center of the neck flat plates. The posterior region of the neck is characterized by 15 trichoscalids (tr) arranged radially: eight single (tr_1_) alternating with seven double trichoscalids (tr_2_; Figs 1, 2 and 7). The trichoscalids are all flat, with a central ridge, and possess serrated margins with hairs and a blunt tip. The double trichoscalids are composed of two separate appendages, an upper appendage and a lower appendage. The upper appendage is longer (ventrolateral pairs) or slightly longer (lateral pairs) than the lower appendage, which has approximately the same length as a single trichoscalid. Both the single and each of the double trichoscalids protrude from a single trichoscalid plate (tp) with a trapezoid shape; the basal plates of the midventral pair are slightly smaller (Figs 2 and 7D). Moreover, each ventrolateral single trichoscalid is further characterized by having two sclerotized plates located anteriorly to their trichoscalid plate. Notably, the basal plates of the upper appendages of the double trichoscalids are characterized by a short sensory organ with serrated (toothed) margins and an anteroproximally situated pore, which is only seen by SEM (to; Figs 1D, 2 and 7D). The trichoscalid sensory organ is situated at the most anterior margin of the upper appendage basal plate. The sensory organs of the ventral double trichoscalid plates are slightly shorter than the ventrolateral ones. The putative sensory organs of the dorsal side could not be observed neither by light nor scanning electron microscopy; their presence/absence remains thus to be determined. In addition, the plate of each midventral single trichoscalid has a large, medially situated pore that is approximately twice the size as the pore on the trichoscalid sensory organs.

Summing up, the number of introvert and neck appendages (cs = clavoscalids, ss = spinoscalids, tp = trichoscalid plates, tr = trichoscalids) is given in the following formula (Fig 8):

$$\text{Introvert}:8\;\text{cs},\;9{\;\text{sr}}_{2},\;7{\;\text{sr}}_{3},\;8+8{\;\text{sr}}_{4},\;30{\;\text{sr}}_{5},\;30{\;\text{sr}}_{6},\;30{\;\text{sr}}_{7},\;30{\;\text{sr}}_{8};\;30{\;\text{sr}}_{9};$$

$$\text{Neck}:7\text{a}+8\text{b}\;\text{bp};\;8+14\;\text{tp},\;8{\;\text{tr}}_{1}(\text{single})+7{\;\text{tr}}_{2}(\text{double}).$$

*Thorax*. Short, enclosed in its own cuticle, surrounded externally by the lorica, and without any appendages or segments.

*Abdomen*. Enclosed in a lorica (lo; Figs 1–7) composed of six cuticularized plates with honeycomb sculpture: one ventral, two ventrolateral, two dorsolateral and one dorsal. Each plate possesses two robust anterior spikes (sp) except for the dorsal plate, which bears four anterior spikes. In total, the anterior edge of the lorica thus has 14 large spikes of equal length, except for one larger spike on each of the dorsolateral plates. In addition, a small midventral spike is present, located between the two large anterior spikes of the ventral plate (Figs 1D, 2, 7C and 7D). The total number of anterior spikes is thus 15.

Notably, the dorsal plate is characterized by two narrow, longitudinal stripes (ls) spanning laterally along its anterior two thirds (Figs 1B and 3–7G). These longitudinal stripes appear as surface ridges and their presence gives the impression that the dorsal plate is partially subdivided into three subplates.

A cluster of four flosculi (fl), arranged in a rectangular pattern, is present on each of the dorsolateral plates (dlp). These flosculi are characterized by 5–6 microvilli-like structures covered with cuticle (Fig 7F and 7G). Additionally, a single smaller flosculum, with only 2–3 microvilli-like structures, is found on the dorsal plate.

The postero-dorsal region of the lorica consists of an anal field (af, Figs 1A, 2, 4A and 6A). More specifically, this anus-gonopore region is characterized, both in males and females, by a small anal cone (Figs 3 and 7G) and a pair of putative gonopores situated posteriorly on the dorsolateral plates (go?; Figs 3 and 4E). Each of the latter plates is furthermore characterized by a cuticularized crest (cc) that spans internally between the putative gonopores and the posterior end of the lorica (Fig 4A). On the ventral side, a large pore (gland outlet?) is situated posteriorly on each margin of the ventral plate (gl?; Figs 2 and 4D) of both males and females.

*Internal anatomy (*Figs 2 *and* 4–6*)*. A long annulated buccal tube (bu) extends between the mouth aperture (mo) and the pharyngeal bulb (pb; Figs 2, 4A, 4B, 5 and 6D). Posteriorly, the pre-pharyngeal armature is characterized by the presence of three buccal furcae arranged radially, which anchor retractor muscles (not shown). In females, the abdominal region is characterized by the presence of two ovaries (ov; Fig 6A), each of which may contain oocytes. Males have two large testes (te) containing spermatozoa (sz; Fig 6C).

**Fig 9:**
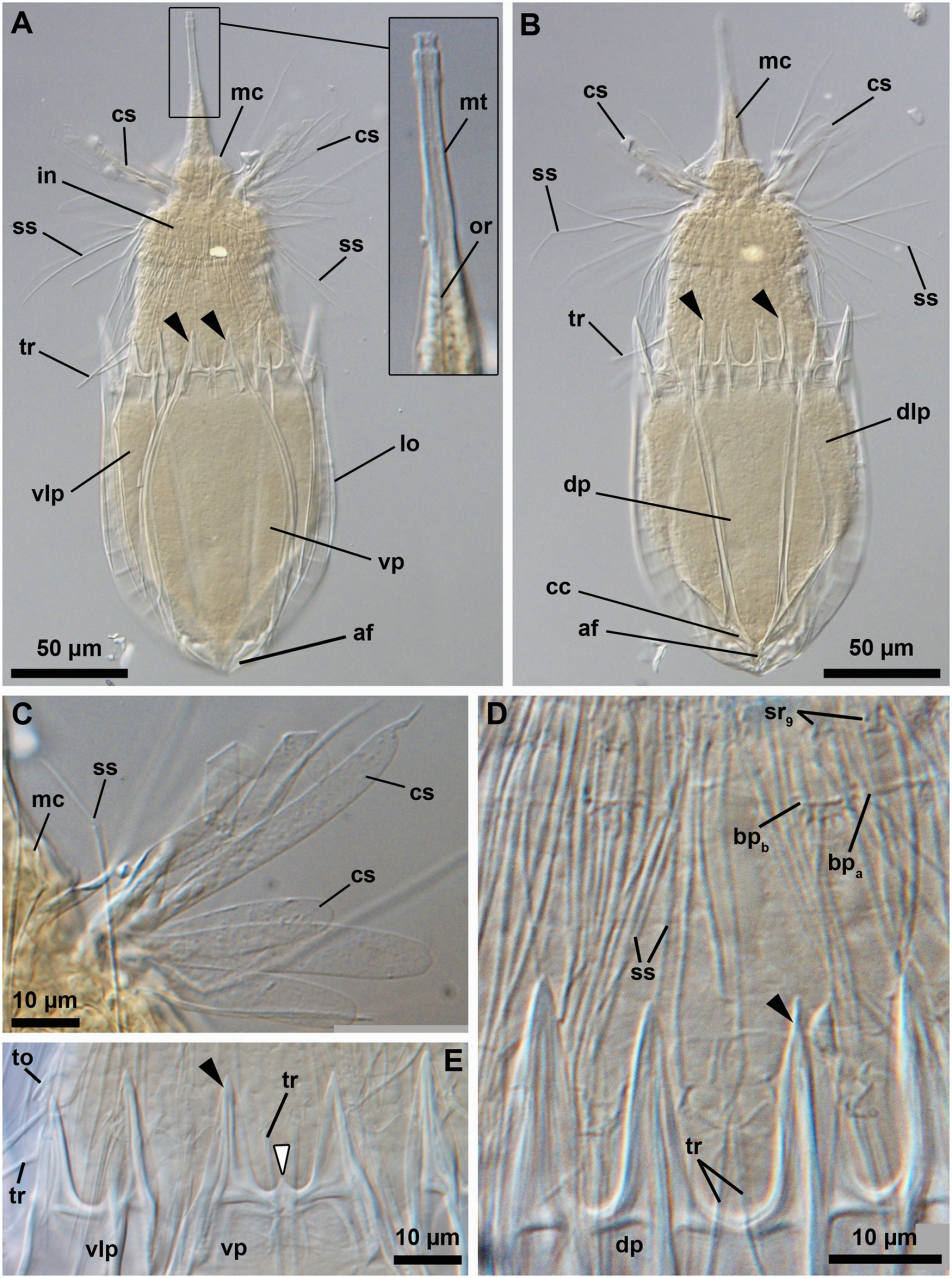
Light micrographs of the holotypic adult male of *Nanaloricus mathildeae* sp. nov. Anterior faces up in all aspects. (A) Ventral view of the extended specimen. Black arrowheads indicate anterior spikes. The inset shows a detail of the mouth tube (mt), which is not fully extended. (B) Dorsal view of the specimen. (C) Close-up of multiform clavoscalids (cs). (D) Close-up of the introvert and anterior spikes of the dorsal plate. Note the presence of basal plates of type a and b (bp_a/b_), and spinoscalids of 9^th^ row (sr_9_); (E) Close-up of the anterior spikes of the ventral and ventrolateral plates. White arrowhead points to the small midventral anterior spike. Abbreviations: af, anal field; cc, cuticularized crest; cs, clavoscalid; dlp, dorsolateral plate; dp, dorsal plate; in, introvert; lo, lorica; mc, mouth cone; mt, mouth tube; or, oral ridge; ss spinoscalid; to, trichoscalid sensory organ; tr, trichoscalid; vlp, ventrolateral plate; vp, ventral plate.

**Fig 10:**
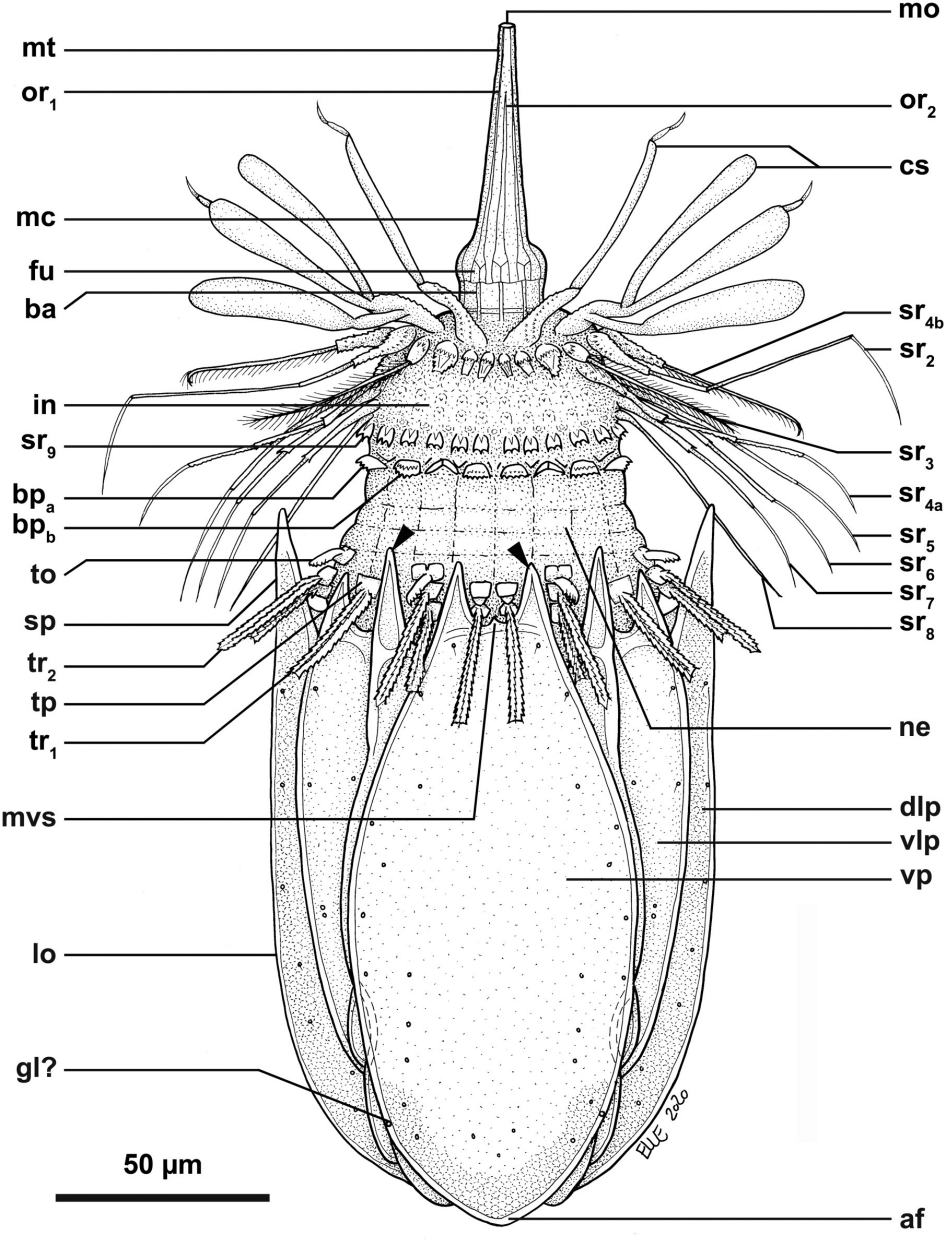
Line art drawing of the adult male habitus of *Nanaloricus mathildeae* sp. nov. Ventral view, anterior faces up. Note that only a selected number of scalids from rows 1 to 8 are represented for clarity. Abbreviations: af, anal field; ba, mouth cone bar; bp_a/b_, basal plates of type a and b; cs, clavoscalid; dlp, dorsolateral plate; fu, oral furca; gl?, putative gland outlets; in, introvert; lo, lorica; mc, mouth cone; mo, mouth aperture; mt, mouth tube; mvs, midventral anterior spike; ne, neck; or_1/2_, oral ridges of type 1 and 2; sr_2–9_, spinoscalids of 2^nd^ to 9^th^ row (including sr_4a/b_, i.e. spinoscalids of 4^th^ row of type a and b); sp (and black arrowheads), anterior spike; to, trichoscalid sensory organ; tp, trichoscalid plate; tr_1_, single trichoscalid; tr_2_, double trichoscalid; vlp, ventrolateral plate; vp, ventral plate.

**Fig 11:**
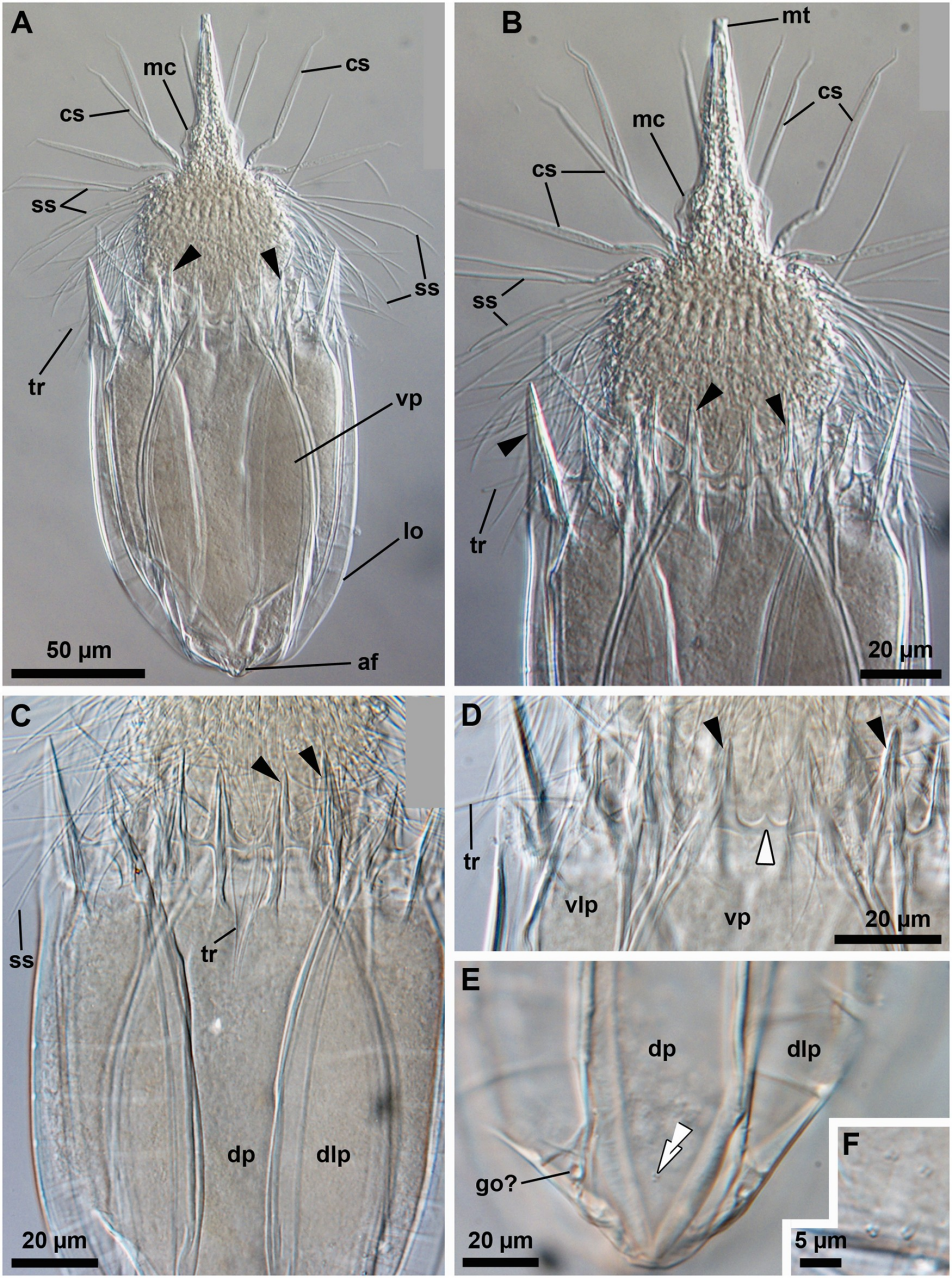
Light micrographs of the allotypic adult female of *Nanaloricus mathildeae* sp. nov. Anterior faces up in all aspects. (A) Ventral view of the extended specimen. Note that the mouth tube is not fully extended. Black arrowheads indicate anterior spikes. (B) Close-up of anterior body half, ventral view. (C) Introvert, neck and anterior region of the abdomen, dorsal view. (D) Close-up of anterior spikes of the ventral and ventrolateral plates. White arrowhead points to the very small midventral anterior spike. (E) Close up of the posterior region of the lorica, dorsal view. Note the putative gonopores (go?) located on the posteriormost region of the dorsolateral plate. Double arrowheads point to the small flosculum situated on the dorsal plate. (F) Detail of a cluster of flosculi arranged in a rectangular pattern on the right dorsolateral plate. Abbreviations: af, anal field; cs, clavoscalid; dlp, dorsolateral plate; dp, dorsal plate; lo, lorica; mc, mouth cone; mt, mouth tube; ss spinoscalid; tr, trichoscalid; vlp, ventrolateral plate; vp, ventral plate.

**Fig 12:**
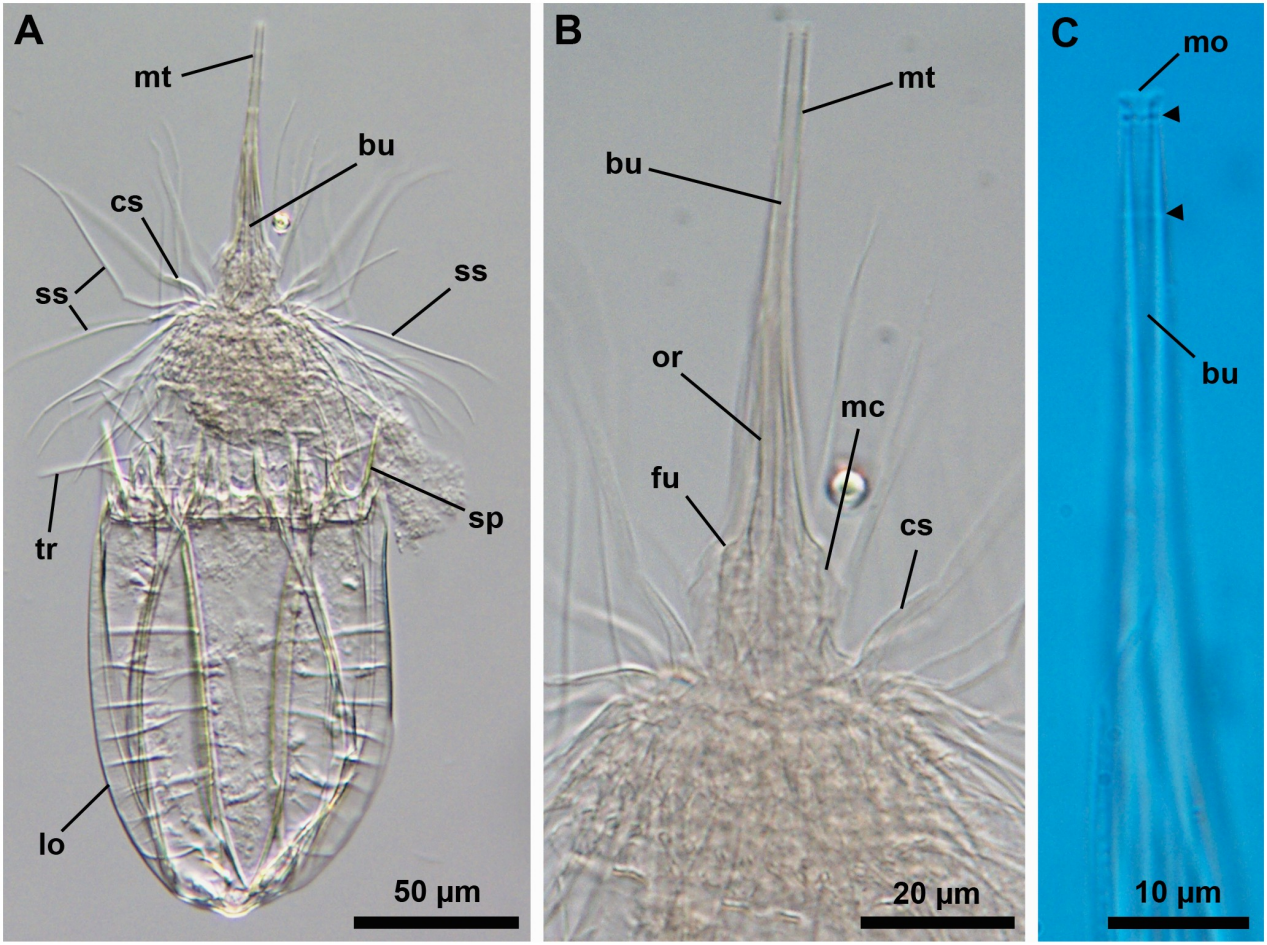
Light micrographs of a paratypic adult female of *Nanaloricus mathildeae* sp. nov. Anterior faces up in all aspects. (A) Overview of the fully extended specimen. Note that the mouth tube is fully extended. (B) Close up of the anterior region of the body. (C) Detail of the anteriormost region of the mouth tube. Short, black arrowheads point to ring-like thickenings of the fully extended mouth tube. Abbreviations: bu, buccal tube; cs, clavoscalids; fu, oral furca; lo, lorica; mc, mouth cone; mo, mouth aperture; mt, mouth tube; or, oral ridge; sp, anterior spike; ss spinoscalid; tr, trichoscalid.

**Fig 13:**
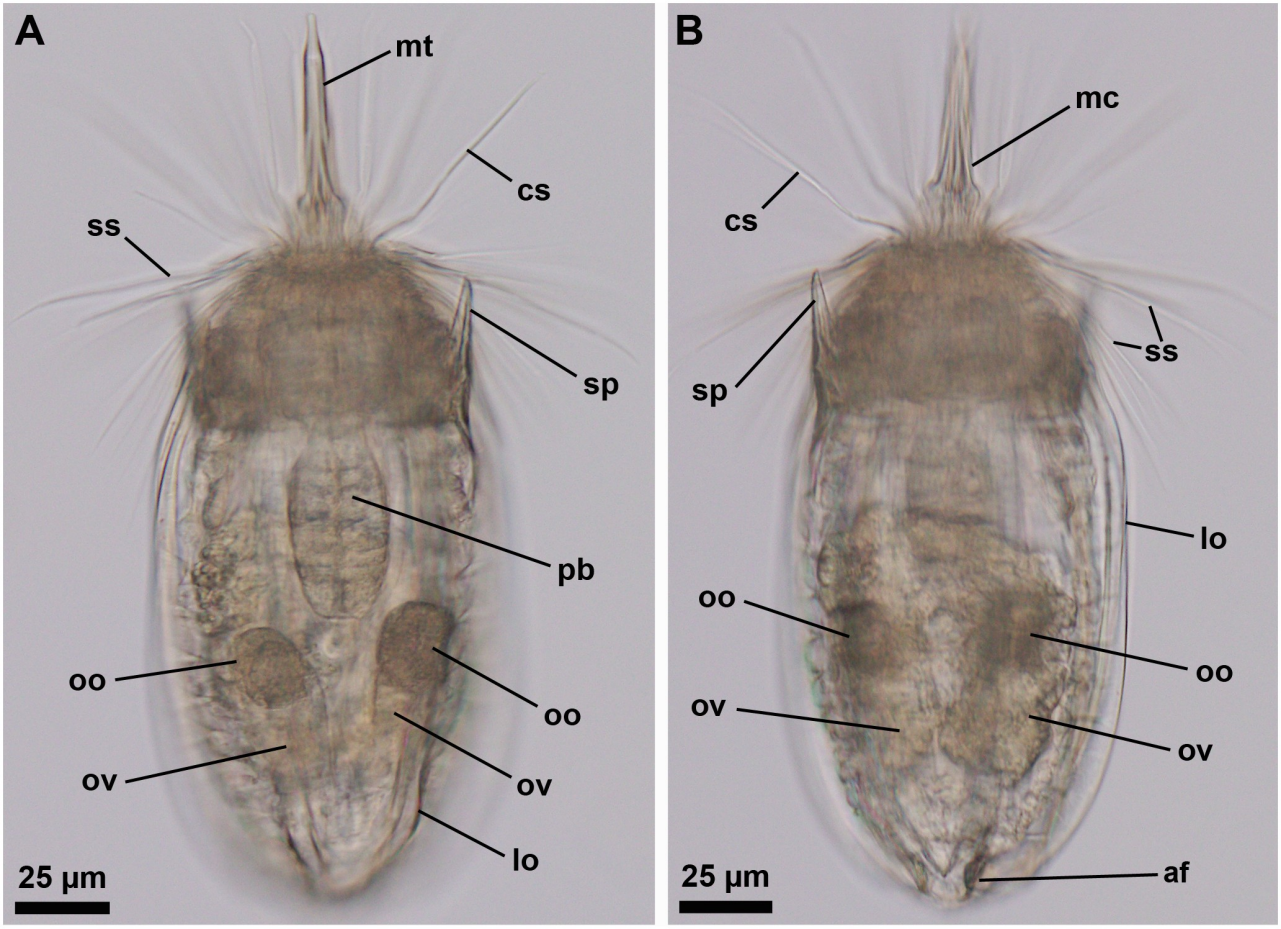
Light micrographs of a live adult female of *Nanaloricus mathildeae* sp. nov. Anterior faces up in both aspects. Note the video sequence of this animal in the S1 Video. (A and B) Overview of the body with partly extended introvert and mouth cone. Internally, note the presence of a large oocyte (oo) within each ovary (ov) in the abdomen. Note also the shape and relative position of the pharyngeal bulb (pb). Abbreviations: af, anal field; cs, clavoscalid; lo, lorica; mc, mouth cone; mt, mouth tube; sp, anterior spike; ss spinoscalid.

**Fig 14:**
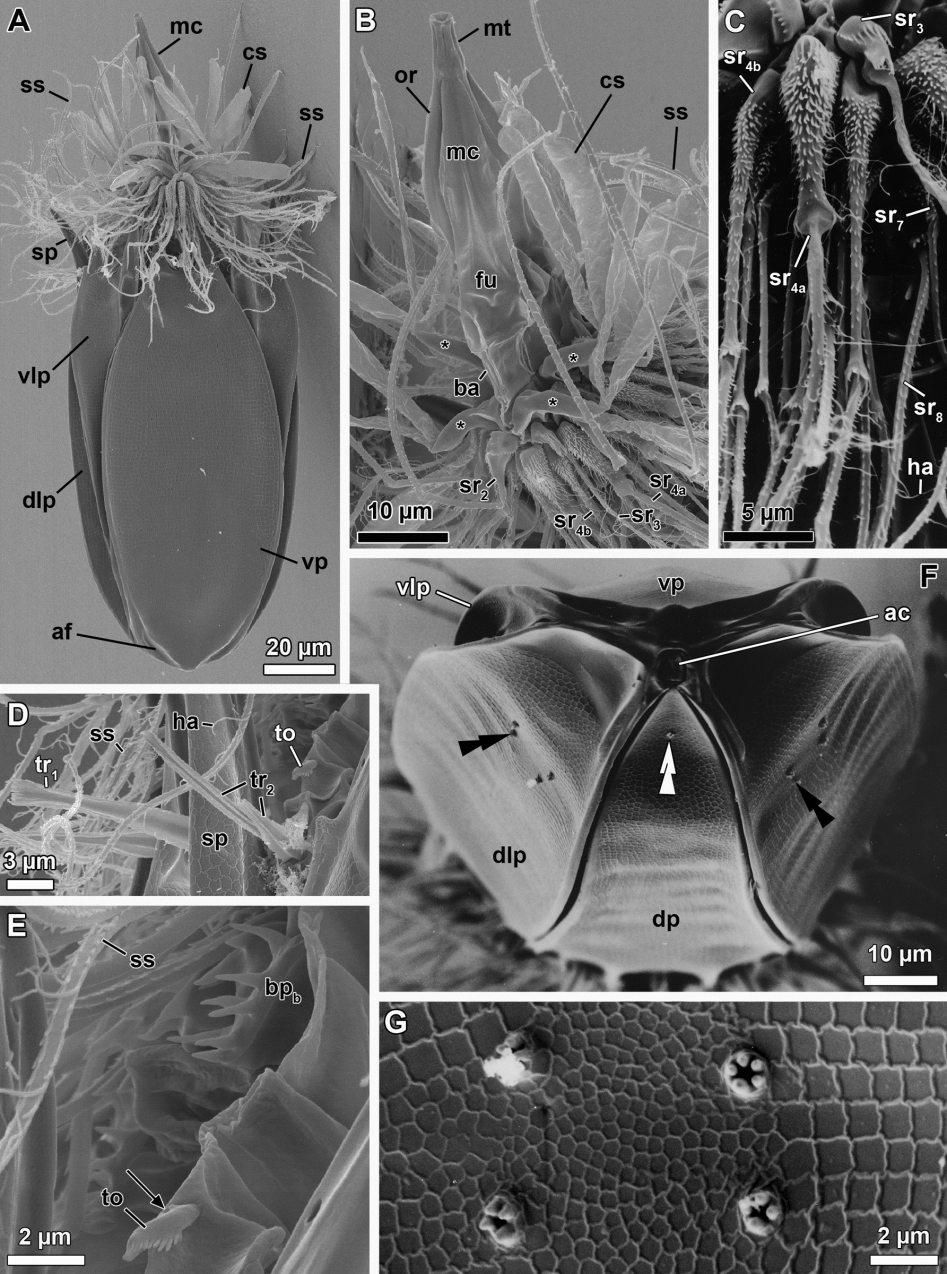
Scanning electron micrographs of a paratypic adult male of *Nanaloricus mathildeae* sp. nov. (A) Overview of the extended specimen, ventral view, anterior faces up. (B) Close up of the anterior body region. Note the broad, multiform clavoscalids (cs). Clavoscalid bases are marked with asterisks (*). (C) Close up of spinoscalids from different rows, each with distinct gross morphology. Note the numerous long hairs (ha) scattered along the length of the various spinoscalids. (D) Close up of a dorsolateral single trichoscalid (tr_1_) and a midlateral (right-hand side) double trichoscalid (tr_2_). Note the relatively small sensory organ (to) of the double trichoscalid. (E) Close up of the trichoscalid sensory organ with a small pore (black arrow). Note also a type b basal plate (bp_b_) of the anterior neck region. (F) Overview of the posterodorsal region of the lorica. Black double arrowheads indicate the cluster of flosculi situated on each dorsolateral plate, while the white double arrowheads point to the small flosculum of the dorsal plate. (G) Detail of a dorsolateral cluster of flosculi arranged in a rectangular pattern. Abbreviations: ac, anal cone; af, anal field; ba, mouth cone bar; cs, clavoscalid; dlp, dorsolateral plate; dp, dorsal plate; fu, oral furca; mc, mouth cone; mt, mouth tube; or, oral ridge; ss, spinoscalid; sp, anterior spike; sr_2-4a/b,7,8_, spinoscalid of 2^nd^ to 4^th^ (type a and b), 7^th^ and 8^th^ rows; vlp, ventrolateral plate; vp, ventral plate.

***Nanaloricus mathildeae*** sp. nov.

(Figs 8–14, S1 Video).

urn:lsid:zoobank.org:act: 953ADF05-F046-4855-A2F8-82F588202999

#### Material examined

*Holotype (*Fig 9*)*. Adult male collected on 8 April 2004 at the type locality at ca. 50 m water depth, mounted in glycerin on a glass slide, and deposited at the Natural History Museum of Denmark under accession number NHMD-678028.

*Allotypic paratype (*Fig 11*)*. Adult female collected on 7 February 2013 at the type locality at ca. 45 m water depth, mounted in glycerol on a glass slide, and deposited at the Natural History Museum of Denmark under accession number NHMD-678039.

*Paratypes*. 23 adults (6 males, 17 females), 2 tentatively assigned postlarvae and 2 putative postlarval exuvia collected at the type locality between 12 July 1985 and 15 May 2013 at 43–55 m water depths. The 27 paratypic specimens are mounted in glycerin or Fluoromount-G on glass slides, deposited at the Natural History Museum of Denmark under the access numbers NHMD-678022 to NHMD-678027, NHMD-678030, NHMD-678032 to NHMD-678038, NHMD-678040 to NHMD-678051 and NHMD-678053. The specimen registered with access number NHMD-678044 in shown in Fig 12. In addition, an adult male collected at the type locality on 12 July 1985, at ca. 55 m water depth, and mounted on an SEM stub (NHMD-866005) was analyzed for comparative purposes (Fig 14).

*Additional reference material (*Fig 13, S1 Video*)*. In addition to the fixed material examined, four non-type specimens (all females), collected at the type locality in April 2019 and August 2020, were observed and photographed alive. Two of these animals were also video recorded alive.

*Habitat and distribution*. Marine sediments composed of clean shell gravel at type locality.

*Type locality*. Trezen ar Skoden, Roscoff, France, (48°45’55”N, 04°06’45”E).

*Etymology*. The species is named after Mathilde Møbjerg Boslev Kristensen, who is granddaughter and niece to the middle author and last author, respectively.

The following description will solely focus on adult specimens, as postlarvae have only been tentatively assigned to species (see section below: *"Notes on the postlarvae found at Trezen ar Skoden"*).

#### Diagnosis

*Adults*. (1) mouth cone with a telescopic mouth tube and 8 oral ridges of different length. Oral ridges are characterized by posterior sclerotized oral furca and are preceded by cuticularized bars; (2) introvert with 9 rows of scalids; (3) first row with eight clavoscalids that are different between males (multiform, very broad to slender, all branched except for the midventral pair) and females (four-segmented, slender, unbranched); (4) second row with 9 four-segmented, leg-like spinoscalids; (5) third row with 7 two-segmented, feather-like scalids; (6) fourth row with 16 spinoscalids of two types: 8 two-segmented, leg-like scalids (type A) alternate with 8 two-segmented scalids with a feather-like distal segment (type B); (7) fifth to seventh rows all similar, each row with 30 leg-like, three-segmented scalids; (8) eighth row with 30 very long unsegmented spinoscalids whith a small conical base; (9) ninth row with 30 small, teeth-like scalids with four cuspid-like protrusions; (10) neck with 8 single trichoscalids alternating with 7 double trichoscalids, with both the single and each of the double trichoscalids protruding from a single trichoscalid plate; (11) short sensory organ located anteroproximally to the ventral and ventrolateral double trichoscalids, characterized by serrated margins, anteroproximal pore and a double-square plate; (12) lorica composed of six cuticular plates, with honeycomb sculpturing and bearing 14 large anterior spikes and a very small midventral spike; (13) 9 flosculi (1 dorsal and 4 pairs laterodorsal) present posteriorly on the dorsal side of the lorica (dorsal and dorsolateral plates); (14) posterior region of the lorica characterized by a small anal cone and two pairs of pores: one dorsolateral (gonopores?) and one ventral (gland outlets?).

#### Description

*Body (*Figs 9–14*)*. Divided into head (mouth cone and introvert), neck, thorax, and abdomen. The holotypic adult male (Fig 9) is 283 μm long, including the mouth cone, and 103 μm wide.

*Mouth cone (mc*; Figs 9–14*)*. Long (76 μm long in the holotype), narrow, with three distinct sections. The first, most proximal section is short, broad and characterized by eight large, sclerotized oral furcae (fu) of identical structure; each furca is posteriorly preceded by a cuticularized bar (ba; Figs 10 and 14B). The furcae and the bars are arranged radially. The middle section is long, conical and characterized by a wall reinforced by eight oral ridges of different length (Fig 10). The four primary oral ridges (or_1_) are long and span the whole length of the middle section, while the four secondary oral ridges (or_2_) are shorter and span only the posterior three quarters of the middle section. Each of the oral ridges is a continuation of an oral furca. The distal section of the mouth cone is a telescopic mouth tube that ends in a terminal mouth aperture. The mouth tube can be extended, thereby significantly increasing the total length of the mouth cone (Fig 12). There are no oral stylets.

*Introvert (in*; Figs 9 *and* 10*)*. Round in shape and characterized by nine rows of scalids arranged radially (Figs 8–14).

First row (sr1; Fig 8) with eight clavoscalids (cs) that differ between males and females (Figs 9–11). In females, all clavoscalids are similar and divided into four segments (Fig 11). The first, most proximal segment is relatively short and possesses several long papillae arranged anteriorly. It consists of a conical base that projects distally in a curved, cylindrical shape. The second segment, which is the longest segment of the clavoscalid, is club-shaped. The third segment is very short and thin. The fourth segment is also very short and ends as a spinose tip. In males, the clavoscalids are multiform (Figs 9, 10 and 14). The most ventral pair is exactly as those of the female (Fig 10). The other six clavoscalids each possess a robust base (Fig 14B) that branches off into primary, secondary and tertiary branches. All branches are very broad and flat, though the secondary branch is the broadest one. This secondary branch has a short, relatively thin base and it branches off halfway through the length of the clavoscalid base (Fig 10). The primary branch is in turn segmented, possessing distaly two short, slightly curved segments: a thin one followed by a terminal spine-like segment.

The second row (sr_2_; Fig 8) consists of nine leg-like spinoscalids (ss), each divided into four segments (sr_2_; Fig 10). The first, most proximal segment has a robust, round base with a row of stiff hairs arranged anteriorly (sr_2_; Fig 14B). The base narrows distally and ends as a cylindrical short portion with a few papillae arranged anteriorly in a row on both sides. The second segment is short and cylindrical, with short hairs arranged posteriorly. The third segment is slightly thinner, represents approximately half of the scalid length, and has serrated margins with several thin hairs. The fourth, most distal segment is slightly curved, terminates as a spinose tip and represents one quarter of the scalid length.

The third row (sr_3_; Fig 8) is composed of seven short, two-segmented feather-like spinoscalids (Figs 10, 14B and 14C). The proximal segment has a swollen, round base that narrows distally and bears two rows of papillae. The distal segment possesses numerous thick hairs and a small hook-shaped tip.

The fourth row (sr_4_; Fig 8) consists of 16 spinoscalids of two types: eight type A leg-like spinoscalids (sr_4a_) alternating with eight type B spinoscalids (sr_4b_) with thick hairs that are only seen by SEM (Figs 10, 14B and 14C). The type A spinoscalids are divided into four segments. The first, most proximal segment has a short, round base that narrows distally and forms a relatively long, cylindrical region characterized by several minute papillae (well discernible by SEM, Fig 14B and 14C). This segment represents slightly more than one fourth of the scalid total length and terminates as a large, round knee. The second segment is thin, slightly shorter than the preceding segment, and possesses serrated margins with a few hairs. The third segment is also thin and possesses serrated margins and a few hairs. The fourth, most distal segment lacks the hairs and terminates as a thin tip. Both the third and fourth segments have approximately the same size as the second segment. The type B spinoscalids are two-segmented. The proximal segment is a short, slightly broad base that narrows distally and possesses several short papillae. The distal segment is thin and characterized by numerous thick hairs arranged posteriorly along the segment.

The fifth to seventh rows (sr_5-7_; Fig 8) are each composed of 30 three-segmented, leg-like spinoscalids (Figs 10 and 14C). Each of the three segments comprise approximately one third of the total scalid length. The first, most proximal segment has a short, conical base that narrows distally and forms a cylindrical region with serrated margins that terminates in a small knee bearing papillae. The second and third, most distal segments are both thin; the latter terminates in a slightly curved spine-like tip. These two most distal segments have finely serrated margins and several long hairs scattered along their length, which are only seen by SEM (Fig 14C).

The eighth row (sr_8_; Fig 8) consists of 30 unsegmented whip-like spinoscalids with a small conical base (Figs 10 and 14C). These scalids terminate as a thin tip.

The ninth row (sr_9_) consists of 30 very short scalids (Figs 9D and 10). Each of these scalids possesses an oval anterior edge and four cuspid-like protrusions, of which three extend backwards from the posterior edge and one from the anterior edge.

*Neck (ne*; Figs 8 *and* 10*)*. Composed of at least five rows of neck flat plates divided into columns by several longitudinal folds (Fig 10). The anterior neck region is characterized by a row of conspicuous plates situated immediately under the ninth row of spinoscalids (Figs 9D, 10 and 14E). Specifically, this row consists of seven triangular plates (bp_a_), which alternate with eight trapezoid plates (bp_b_). Both types are characterized by two transverse rows of teeth. Each basal plate is situated in the center of the neck flat plates. The posterior region of the neck is characterized by 15 trichoscalids (tr; Fig 9) arranged radially: eight single (tr_1_) alternating with seven double trichoscalids (tr_2_) (Figs 10 and 14D). The trichoscalids are all flat and with a central ridge, serrated margins with few hairs and terminate in either a blunt or a pointy tip. The upper appendages of the double trichoscalids are longer than the lower appendages. In addition, the upper appendages have a pointy tip, while the lower appendages have a blunt tip. The single trichoscalids are broader than the double trichoscalids, and possess a unique blunt end composed of several (ca. 14?) robust finger-like processes. On both sides of the single trichoscalids, the most lateral finger-like process is longer and thus extends further than the more medial ones (only seen by SEM; Fig 14D). Both the single trichoscalids and each of the two appendages composing the double trichoscalids protrude from a single trapezoid trichoscalid plate (tp). However, each single trichoscalid composing the midventral pair protrudes from a pentagonal plate. Each of these midventral single trichoscalids is further characterized by two sclerotized plates, one large and one small, located anteriorly to their trichoscalid plate. A short sensory organ is located anteroproximally to the ventral and ventrolateral double trichoscalids (to; Figs 10, 14D and 14E). The trichoscalid sensory organ protrudes from a plate with a double-square shape (Fig 10), and is characterized by serrated (toothed) margins and an anteroproximally situated pore (Fig 14E). The putative sensory organs of the dorsal side could not be observed neither by light nor scanning electron microscopy; their presence/absence remains thus to be determined.

Summing up, the number of head and neck appendages (cs = clavoscalids, ss = spinoscalids, tp = trichoscalid plates, tr = trichoscalids) is given in the following formula (Fig 8):

$$\text{Introvert}:8\;\text{cs},\;9{\;\text{sr}}_{2},\;7{\;\text{sr}}_{3},\;8+8{\;\text{sr}}_{4},\;30{\;\text{sr}}_{5},\;30{\;\text{sr}}_{6},\;30{\;\text{sr}}_{7},\;30{\;\text{sr}}_{8};\;30{\;\text{sr}}_{9};$$

$$\text{Neck}:7\text{a}+8\text{b}\;\text{bp};\;8+14\;\text{tp},\;8{\;\text{tr}}_{1}(\text{single})+7{\;\text{tr}}_{2}(\text{double}).$$

*Thorax*. Short, surrounded by the lorica and without any appendages or segmentation.

*Abdomen*. Enclosed in a lorica (lo; Figs 9–14) composed of six cuticularized plates with a honeycomb sculpture: one ventral, two ventrolateral, two dorsolateral and one dorsal. The latter plate possesses four anterior spikes (sp), while each of the other plates bears only two. In total, the anterior edge of the lorica is characterized by 14 large spikes of equal length, except for one larger spike on each of the dorsolateral plates. In addition, a very small midventral anterior spike is located between the two large anterior spikes of the ventral plate. The total number of anterior spikes is thus 15 (Figs 9E, 10 and 11D).

A cluster of four flosculi (Figs 11F and 14G) is arranged in a rectangular pattern on each of the dorsolateral plates (dlp; Fig 14F). These flosculi are characterized by 4–5 microvilli-like stuctures covered with cuticle (Fig 14G). Additionally, one smaller flosculum with 3 microvilli-like structures is located posteriorly on the dorsal plate (Figs 11E and 14F).

The postero-dorsal region of the lorica consists of an anal field (af, Figs 9A, 9B, 10 and 11A). In both males and females this anus-gonopore region is characterized by a small anal cone (Fig 14F) and, close to it, a pair of putative gonopores (go?; Fig 11E). These large pores are located on the posterior region of the dorsolateral plates. A cuticularized crest (cc, Fig 9B) spans between each of the putative gonopores and the posterior end of the lorica. On the ventral side, a pair of small pores (gland outlets?) is furthermore located posteriorly on each margin of the ventral plate (gl?; Fig 10).

*Internal anatomy (*Figs 12 *and* 13, S1 Video*)*. A long buccal tube (bu; Fig 12) extends from the mouth aperture (mo; Fig 10)—through the head/thoracic region—to the pharyngeal bulb (pb; Fig 13, S1 Video). The most posterior region of the buccal tube is characterized by a supporting pre-pharyngeal armature, which is composed of three internal buccal furcae (not shown). In females, the abdominal region is characterized by the presence of two ovaries, each of which may contain oocytes. In a female observed alive, each of the two ovaries (ov) was found containing a large oocyte (oo; Fig 13, S1 Video). Males are characterized by two large testes, which contain mature spermatozoa in some specimens (not shown).

*Genus*. *Scutiloricus* gen. nov.

urn:lsid:zoobank.org:act: 590F4BBB-4261-472F-AD76-8272C5FFE3C5

(Type species: *Scutiloricus hugoi* gen. et sp. nov.)

#### Genus diagnosis

*Adults*. (1) mouth cone with 8 oral ridges of different length and characterized by posterior sclerotized oral furca and absence of oral stylets; (2) introvert with 9 rows of scalids; (3) first row with eight clavoscalids that differ between males (multiform, broad and flat, all branched except for the midventral pair) and females (four-segmented, slender, unbranched); (4) second row with 9 four-segmented, leg-like spinoscalids; (5) third row with 7 two-segmented, feather-like scalids; (6) fourth row with 16 spinoscalids of two types: 8 three-segmented, leg-like scalids (type A) alternate with 8 three-segmented spinoscalids with a feather-like distal segment (type B); (7) fifth to seventh rows all similar, each row with 30 leg-like, three-segmented scalids; (8) eighth row with 30 long, unsegmented spinoscalids whith a small conical base; (9) ninth row with 30 oval, plate-like scalids with several minute teeth; (10) neck with 8 single trichoscalids alternating with 7 double trichoscalids, with both the single and each of the double trichoscalids protruding from a single trichoscalid plate; (11) square lorica composed of six cuticular plates with reinforced anterior margins, honeycomb sculpturing and bearing a total of 14 large anterior spikes; (12) anterior spikes with 3–4 transverse cuticular ridges, except for the lateral spikes, which are reinforced internally by thick cuticle with a triangle wave-shape; (13) 10 flosculi (4 dorsolateral pairs and 1 dorsal pair) present posteriorly on the lorica; (14) posterior region of the lorica characterized by a slightly invaginated anal field from which a small anal cone protrudes posteroventrally, flanked by a pair of small spurs; (15) posterior region of the lorica also characterized by two pairs of pores: a dorsolateral (gonopores?) pair and a ventral (gland outlets?) pair; (16) internally, females are characterized by a pair of seminal receptacles (filled with mature spermatozoa) located in the abdomen and postero-dorsally to the ovaries. Larval stages were not found.

*Etymology*. The generic name is composed by the Latin words scuti (= shield) and lorica (corset), masculine gender.

***Scutiloricus hugoi*** gen. et sp. nov.

(Figs 15–19).

**Fig 15:**
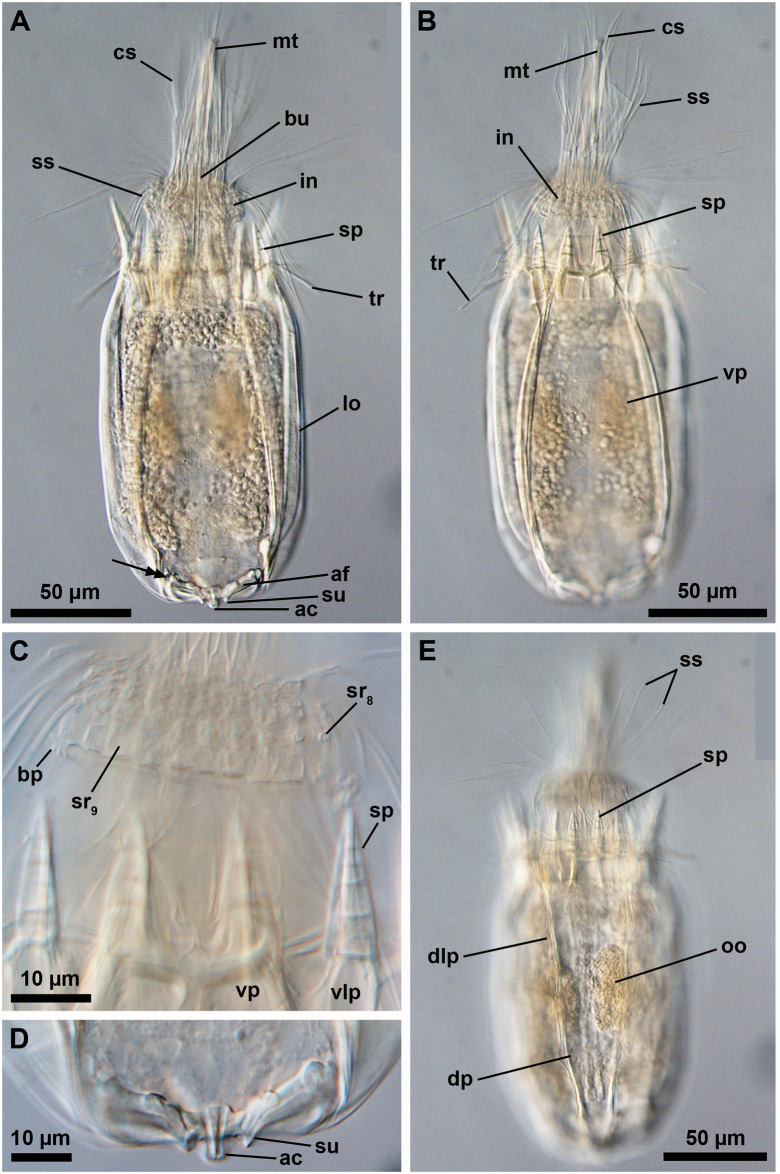
Light micrographs of the holotypic adult female of *Scutiloricus hugoi* gen. et sp. nov. Anterior faces up in all aspects. (A) Overview of the extended specimen, with focus on the internal buccal tube (bu). Double-headed arrow points to large pores (gland outlets?) situated on the posterior region of the ventral plate. (B) Ventral view of the specimen. (C) Close-up of the introvert, neck and anterior region of the lorica, ventral view. Note the fenestrated aspect of the anterior spikes (sp). (D) Close-up of the anal field. (E) Dorsal view of the extended specimen. Abbreviations: ac, anal cone; af, anal field; bp, basal plate; cs, clavoscalid; dlp, dorsolateral plate; dp, dorsal plate; in, introvert; lo, lorica; mt, mouth tube; oo, oocyte; sp, anterior spike; sr_8/9_, spinoscalids of 8^th^ or 9^th^ row; ss spinoscalid; su, anal field spurs tr, trichoscalid; vlp, ventrolateral plate; vp, ventral plate.

**Fig 16:**
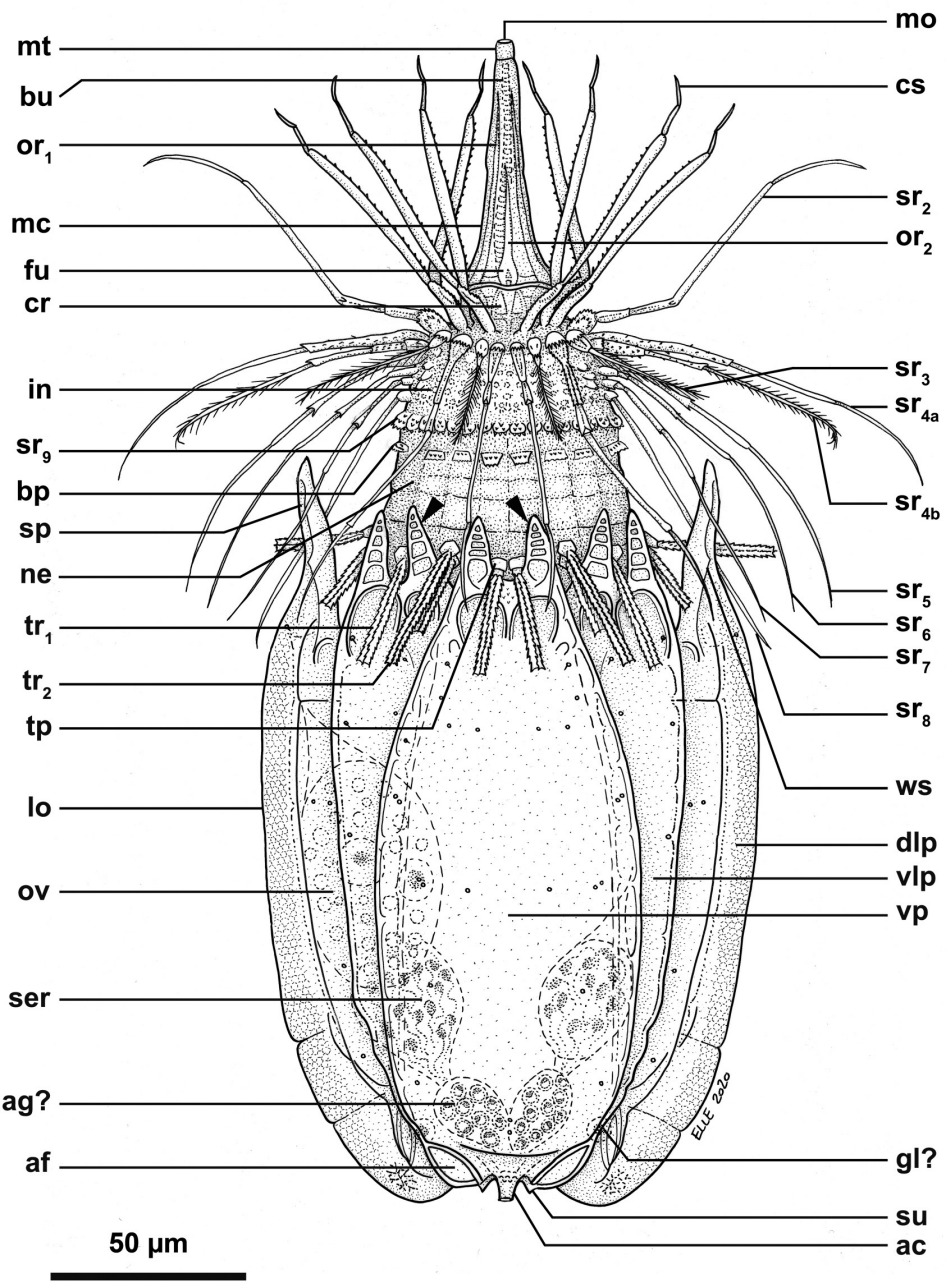
Line art drawing of the adult female habitus of *Scutiloricus hugoi* gen. et sp. nov. Ventral view, anterior faces up. Note that only a selected number of scalids from rows 2 to 8 are represented for clarity. Abbreviations: ac, anal cone; af, anal field; ag?, putative adhesive gland; bp, basal plate; bu, buccal tube; cr, cuticularized triangular ridge; cs, clavoscalid; dlp, dorsolateral plate; fu, oral furca; gl?, putative gland outlet; in, introvert; lo, lorica; mc, mouth cone; mo, mouth aperture; mt, mouth tube; ne, neck; or_1/2_, oral ridges of type 1 and 2; ov, ovary; ser, seminal receptacle; sr_2–9_, spinoscalids of 2^nd^ to 9^th^ row; sp (and arrowheads), anterior spike; su, anal field spurs; tp, trichoscalid plate; tr_1_, single trichoscalid; tr_2_, double trichoscalid; vlp, ventrolateral plate; vp, ventral plate; ws, wave-shaped lateral reinforcement.

**Fig 17:**
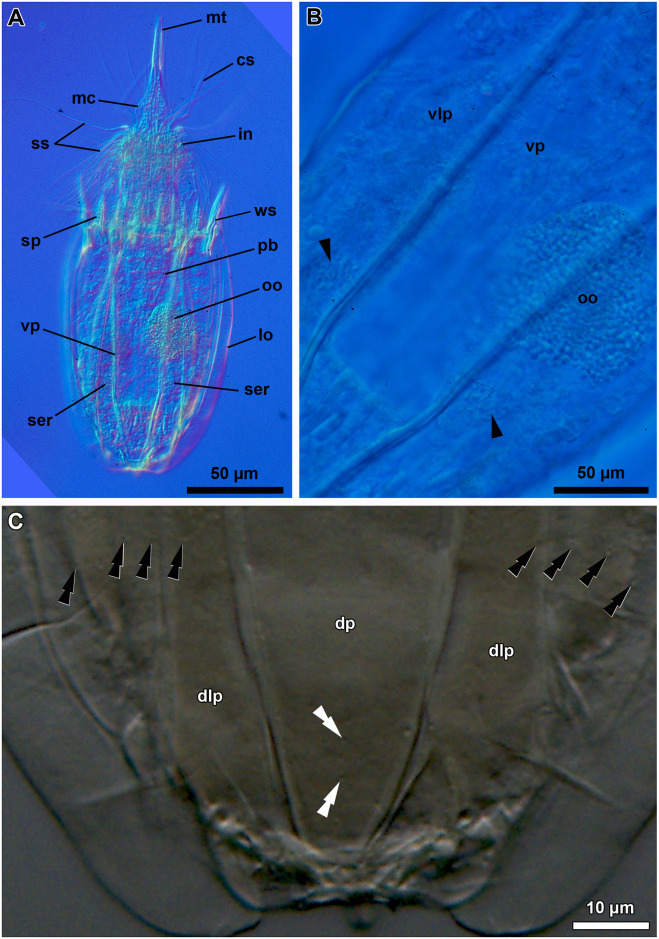
Light micrographs of a paratypic adult female of *Scutiloricus hugoi* gen. et sp. nov. (A) Overview of the fully extended specimen, anterior faces up. Note the presence of a large oocyte (oo) and two seminal receptacles (ser). (B) Close up of the oocyte and the two seminal receptacles (arrowheads). (C) Close up of the posterior region of the lorica. Note the linear arrangement of the four flosculi (black double arrowheads) on each dorsolateral plate (dlp) and the two flosculi (white double arrowheads) located medially on the dorsal plate (dp). Abbreviations: cs, clavoscalids; in, introvert; lo, lorica; mc, mouth cone; mt, mouth tube; sp, anterior spike; ss, spinoscalid; vlp, ventrolateral plate; vp, ventral plate; ws, wave-shaped lateral reinforcement.

**Fig 18:**
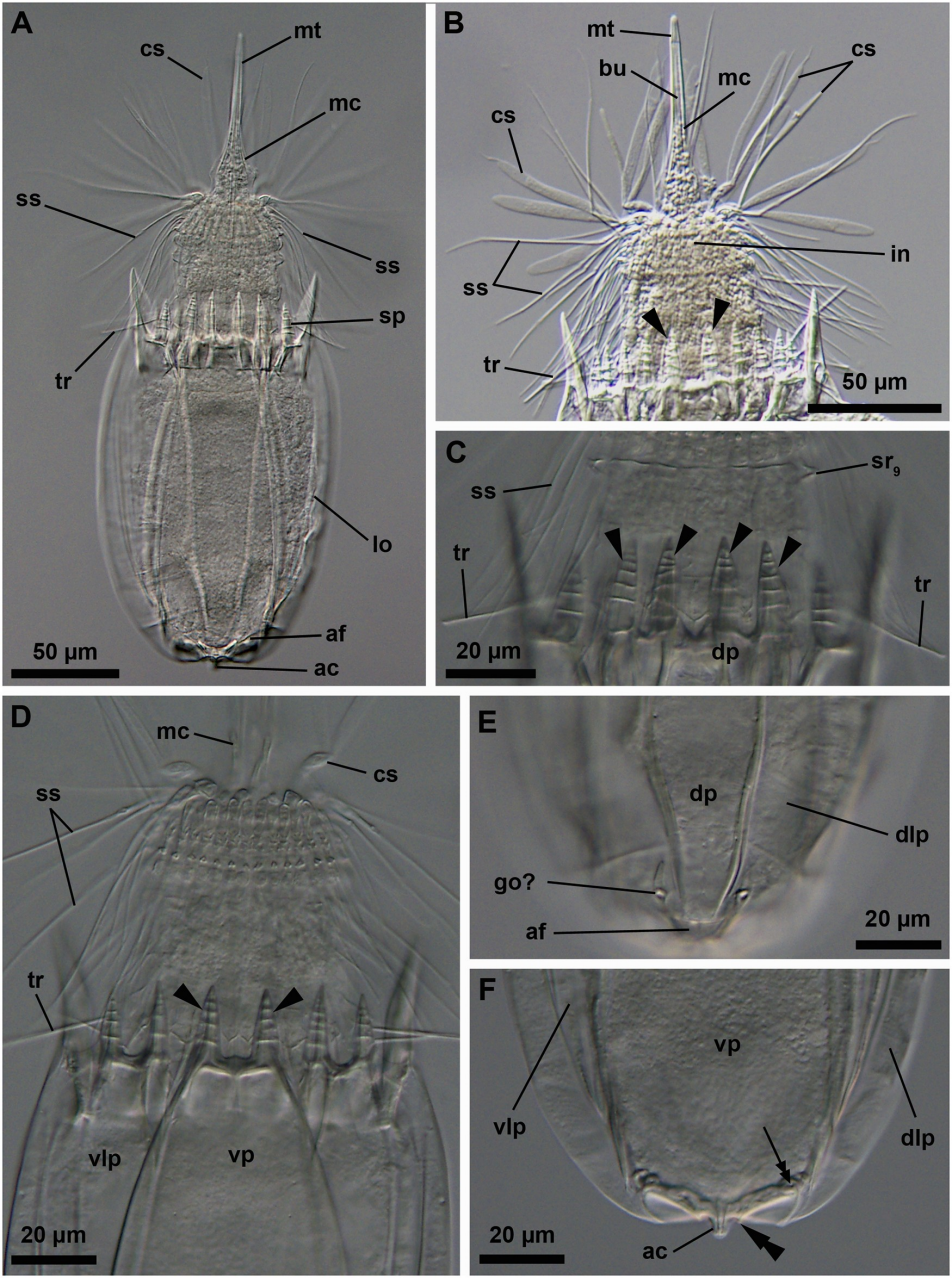
Light micrographs of the allotypic adult male of *Scutiloricus hugoi* gen. et sp. nov. Anterior faces up in all aspects. (A) Overview of the extended specimen, dorsal view. (B) Anterior region of the body, ventral view. (C) Close-up of the anterior spikes of the dorsal and dorsolateral plates. Note the fenestrated aspect of the anterior spikes (arrowheads). (D) Introvert, neck and anterior region of the abdomen; ventral view. (E) Posterior region of the lorica, dorsal view. Note the putative gonopores (go?) located in the posteriormost region of the dorsolateral plates. (F) Posterior region of the lorica, ventral view. Double-headed arrow points to large pore (gland outlet?) situated in the posterior region of the ventral plate. Double arrowheads indicate an anal field spur. Abbreviations: ac, anal cone; af, anal field; bu, buccal tube; cs, clavoscalid; dlp, dorsolateral plate; dp, dorsal plate; in, introvert; lo, lorica; mc, mouth cone; mt, mouth tube; sp and black arrowheads, anterior spike; sr_9_, spinoscalids of 9^th^ row; ss spinoscalid; tr, trichoscalid; vlp, ventrolateral plate; vp, ventral plate.

**Fig 19:**
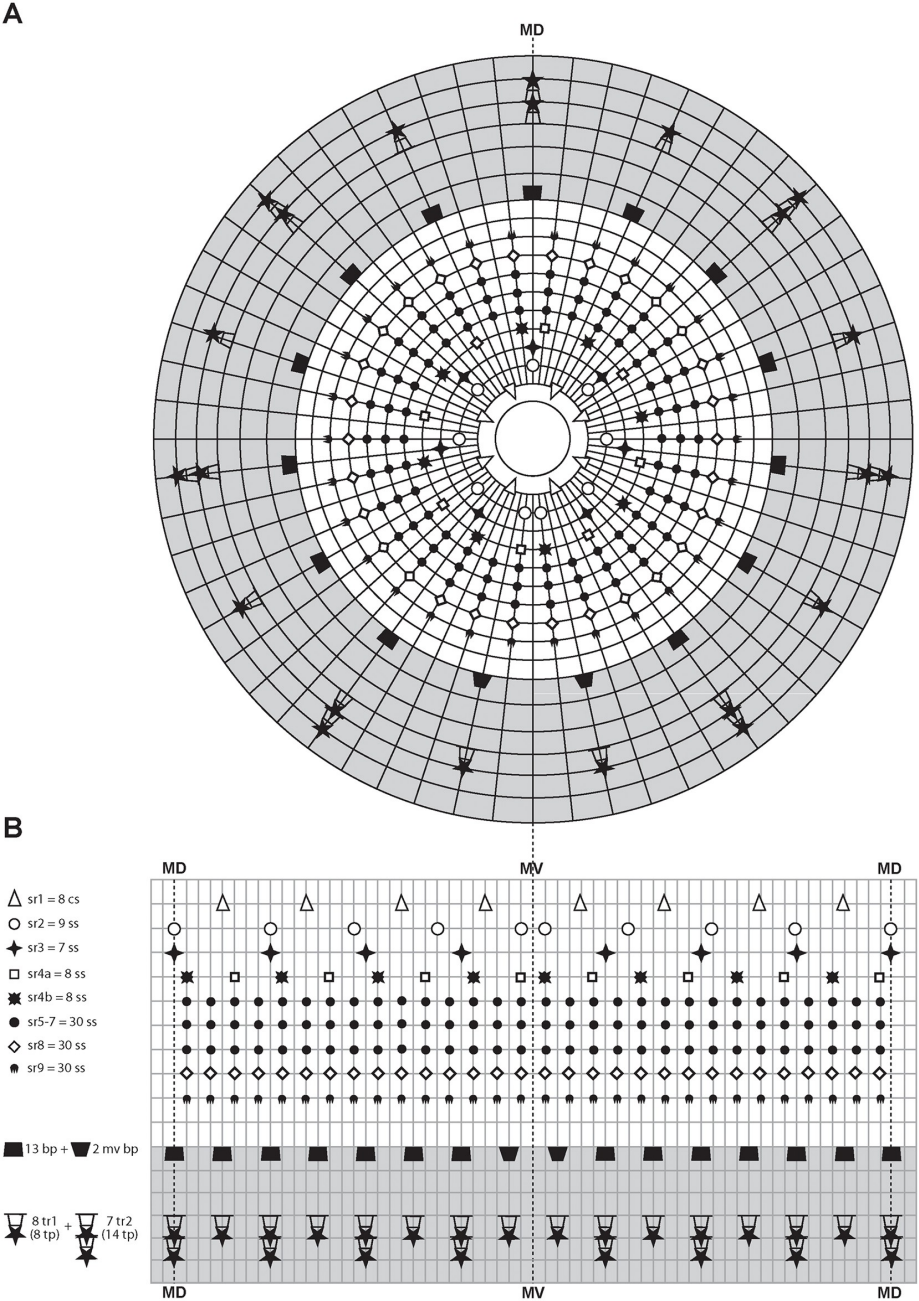
Schematic diagrams of the distribution of introvert and neck appendages in adult forms of *Scutiloricus hugoi* gen. et sp. nov. Note that for the sake of clarity sexual dimorphism of the clavoscalids is not depicted. The shaded area indicates the neck region. (A) Polar diagram. (B) Planar projection. Abbreviations: bp, basal plate; cs, clavoscalid; MD, middorsal line; MV, midventral line; mv bp, midventral pair of basal plates; sr_1–9_, scalids of 1^st^ to 9^th^ row; ss, spinoscalids; tp, trichoscalid plate; tr_1_, single trichoscalid; tr_2_, double trichoscalid.

urn:lsid:zoobank.org:act: 7636582D-43E3-4F7E-B58A-3543155893C3

#### Material examined

*Holotype (*Fig 15*)*. Adult female collected on 15 May 2013 at the type locality at ca. 50 m water depth, mounted in Fluoromount-G on a glass slide, deposited at the Natural History Museum of Denmark under accession number NHMD-677520.

*Allotypic paratype (*Fig 18*)*. Adult male collected on 7 February 2013 at the type locality at ca. 45 m water depth, mounted in glycerol on a glass slide, deposited at the Natural History Museum of Denmark under accession number NHMD-677519.

*Paratypes*. Include two females mounted in glycerol on glass slides and deposited at the Natural History Museum of Denmark under the access numbers NHMD-677517 (Fig 17) and NHMD-677518. Paratypes were collected at the type locality on 12 July 1985 at 50–55 m water depths.

*Habitat and distribution*. Marine sediments composed of clean shell gravel at type locality.

*Type locality*. Trezen ar Skoden, Roscoff, France, (48°45’55”N, 04°06’45”E).

*Etymology*. The species name is in honour of Hugo Joseph Cornu Neves, the son of first author Ricardo Cardoso Neves.

#### Diagnosis

Same as genus.

#### Description

*Body (*Figs 15–18*)*. Divided into head (mouth cone and introvert), neck, thorax, and abdomen. The holotypic adult female (Fig 15) is 240 μm long, including the mouth cone, and 83 μm wide.

*Mouth cone (mc*; Figs 16–18*)*. Long (62 μm in length in the holotypic adult female), narrow and divided into three distinct sections. The first, most proximal section is short, broad and surrounded by eight large, sclerotized oral furcae (fu) of identical structure (Fig 16). Below the furcae are eight cuticularized triangular ridges (cr). The second, middle section is long, conical and characterized by a wall reinforced by eight oral ridges (or) of different length. The primary oral ridges (or_1_) are long and span almost the entire length of the middle section, while the secondary oral ridges (or_2_) are shorter and span only half of the middle section length. The third, most distal section is a mouth tube that ends with a terminal mouth aperture. There are no oral stylets.

*Introvert (in*, Figs 15–18*)*. Round in shape and characterized by nine rows of scalids arranged radially (Figs 15–19).

First row with eight clavoscalids (sr_1_; Fig 19) that are different between females and males (cs; Figs 15–18). In females, all clavoscalids are identical, slender appendages divided into four segments (Figs 15 and 16). The first, most proximal segment is short and cylindrical, and has several minute teeth arranged antero-distally. The second segment is the longest segment of the clavoscalid and is serrated on the anterior side. The third segment is short and thin. The fourth, most distal segment is short and curved, and ends as a spinose tip. In males, clavoscalids are multiform (Figs 18A and 18B). The morphology of the most ventral pair is exactly as that observed in the female clavoscalids. The other six clavoscalids possess a broad base and are divided into primary, secondary and tertiary branches. All branches are broad and flat, though the secondary branch is slightly broader than the other branches. Both the secondary and tertiary branches are unsegmented. The primary branch is three segmented, possessing two short distal segments: a thin one followed by a terminal, slightly curved segment with a spinose tip (Fig 18B).

The second row (sr_2_; Fig 19) is composed of nine leg-like spinoscalids, which are divided into four segments (Fig 16). The first, most proximal segment has a short, robust base with several minute hairs. The base narrows distally and ends as a cylindrical short region also covered with several minute hairs. The second segment is also short and cylindrical. The third segment represents approximately half of the scalid length. The fourth, most distal segment is curved, with a pointy tip and represents approximately one quarter of the scalid. Noteworthy, the two midventral spinoscalids of the second row are thinner and slightly shorter than all other spinoscalids of the same row.

The third row (sr_3_; Fig 19) consists of seven short, two-segmented spinoscalids (Fig 16). The proximal segment is a short, conical base that narrows distally. The distal segment is relatively long and ends as a thin tip. This segment possesses numerous thick hairs, which gives it a feather-like appearance.

The fourth row (sr_4_; Fig 19) is composed of 16 spinoscalids of two types: 8 type A leg-like spinoscalids (sr_4a_) alternating with 8 type B spinoscalids (sr_4b_) with a hook-shaped tip (Fig 16). The type A spinoscalids are divided into three segments. The proximal segment is a long and relatively broad base, which ends as a small knee. The middle segment is thin and has approximately the same length as the proximal segment. The third segment is slightly shorter than the preceding segments and terminates as a thin tip. The type B spinoscalids are three-segmented as well. The proximal segment is a short, broad base that narrows distally and possesses numerous short hairs. The middle segment is thin and short and terminates as a small knee. The distal segment is thin, characterized by numerous thick hairs and terminates as a hook-shaped tip.

The fifth to seventh rows (sr_5-7_; Fig 19) are each composed of 30 four-segmented, leg-like spinoscalids (Fig 16). The first, most proximal segment is short, with an enlarged base. The two middle segments are long and thin, of which the most proximal one terminates as a knee. The most distal segment is longer and thinner than the preceding two segments (representing approximately one-half of the scalid length) and terminates as a spinose tip.

The eighth row (sr_8_, Fig 19) consists of 30 unsegmented whip-like spinoscalids with a small conical base (Fig 16). These segments terminate as a thin tip.

The ninth row (sr_9_, Fig 19) consists of 30 very short, plate-like scalids with an oval shape and several minute teeth (Figs 15C and 18C).

*Neck (ne*; Figs 16 *and* 19*)*. Appears accordion-shaped due to the presence of several rows (up to six) of neck flat plates, which are divided into columns by longitudinal folds. In the most anterior neck region, a row of 15 small trapezoid basal plates (bp; Fig 16) is situated immediately under the ninth row of spinoscalids (Figs 15C and 16). Thirteen of these basal plates are identical, with the posterior side slightly longer than the anterior side (bp; Fig 19). The two midventral plates, however, have the posterior side shorter than the anterior side (mv bp; Fig 19). All basal plates are characterized by two rows of ca. 3 minute teeth and each plate is situated anteromedially on a neck flat plate. The posterior region of the neck is characterized by 15 trichoscalids (tr) arranged radially: 8 single (tr_1_) alternating with 7 double trichoscalids (tr_2_). The trichoscalids are all flat and characterized by a central ridge, serrated margins and a blunt tip (Figs 15, 16 and 18). The upper appendages of the double trichoscalids are approximately one-fourth longer than those of the lower appendages. The single trichoscalids are slightly shorter than the upper appendages of the double trichoscalids. Both the single and each appendage of the double trichoscalids protrude from a single trichoscalid plate (tp) with a pentagonal shape. However, each single trichoscalid composing the midventral pair protrudes from a rectangular trichoscalid plate.

Summing up, the number of head and neck appendages (cs = clavoscalids, ss = spinoscalids, tp = trichoscalid plates, tr = trichoscalids) is given in the following formula (Fig 19):

$$\text{Introvert}:8\;\text{cs},\;9{\;\text{sr}}_{2},\;7{\;\text{sr}}_{3},\;8+8{\;\text{sr}}_{4},\;30{\;\text{sr}}_{5},\;30{\;\text{sr}}_{6},\;30{\;\text{sr}}_{7},\;30{\;\text{sr}}_{8};\;30{\;\text{sr}}_{9};$$

$$\text{Neck}:13\;\text{bp}+2\;\text{mv}\;\text{bp};\;8+14\;\text{tp},\;8{\;\text{tr}}_{1}(\text{single})+7{\;\text{tr}}_{2}(\text{double}).$$

*Thorax*. Short, surrounded by the lorica; without any appendages or segmentation.

*Abdomen*. Enclosed in a square lorica (lo; Figs 15–18), composed of six cuticularized plates with honeycomb sculpture: one ventral, two ventrolateral, two dorsolateral and one dorsal. The ventral, ventrolateral and dorsolateral plates each possess two robust anterior spikes (sp), whereas the dorsal plate has four. In total, the anterior edge of the lorica thus has 14 large spikes. One of the lateral spikes on each side of the animal is relatively large, whereas the other spikes are of equal length. Except for these larger lateral spikes, all anterior spikes are characterized by 3–4 cuticular transverse ridges, which give the spikes a fenestrated appearance (Figs 15B, 16, 18C and 18D). A midventral short spike is absent. Internally, the larger lateral spikes are characterized by a lateral reinforcement of thick cuticle with a triangle wave-shape (ws; Figs 16 and 17A).

Posteriorly on each of the dorsolateral plates, a cluster of four flosculi (fl) is arranged in a transverse, almost linear pattern (Fig 17C). Additionally, two flosculi are situated on the most posterior region of the dorsal plate. These flosculi are arranged medially along the lorical longitudinal axis (Fig 17C).

The most posterior region of the lorica is a slightly invaginated anal field (af, Figs 15A, 16 and 18A). This posterior anus-gonopore region is composed of several sub-regions (both internal and external) delimited by elevations of the cuticle. A small anal cone (ac; Figs 15D, 16 and 18F) protrudes ventrally from the anal field, flanked on each side by a small spur (su; Figs 15D, 16 and 18F). These anal field spurs are slightly larger in the female (Fig 15A and 15D) as compared to the male (Fig 18A and 18F). On the ventral side, a large pore (gland outlet?) is located posteriorly on each margin of the ventral plate (Fig 18F). A large putative gonopore (go?, Fig 18E) is located dorsally in the posteriormost region of each dorsolateral plate.

*Internal anatomy*. An annulated buccal tube (bu; Figs 15A, 16 and 18) extends between the mouth aperture (mo; Fig 16) and the pharyngeal bulb (pb; Fig 17). Two small, putative adhesive glands (ag?) are located medially in the posteriormost region of the abdomen (Fig 16). Females are characterized by a pair of seminal receptacles located postero-dorsally to the ovaries, which contain the oocytes (oo; Fig 17A and 17B). Mature spermatozoa were found in one of the investigated paratypic females (arrowheads; Fig 17B). Outlets or ducts of the seminal receptacles were not found. Males are characterized by two large testes with mature spermatozoon (not shown).

### Notes on the postlarvae found at Trezen ar Skoden

**Fig 20:**
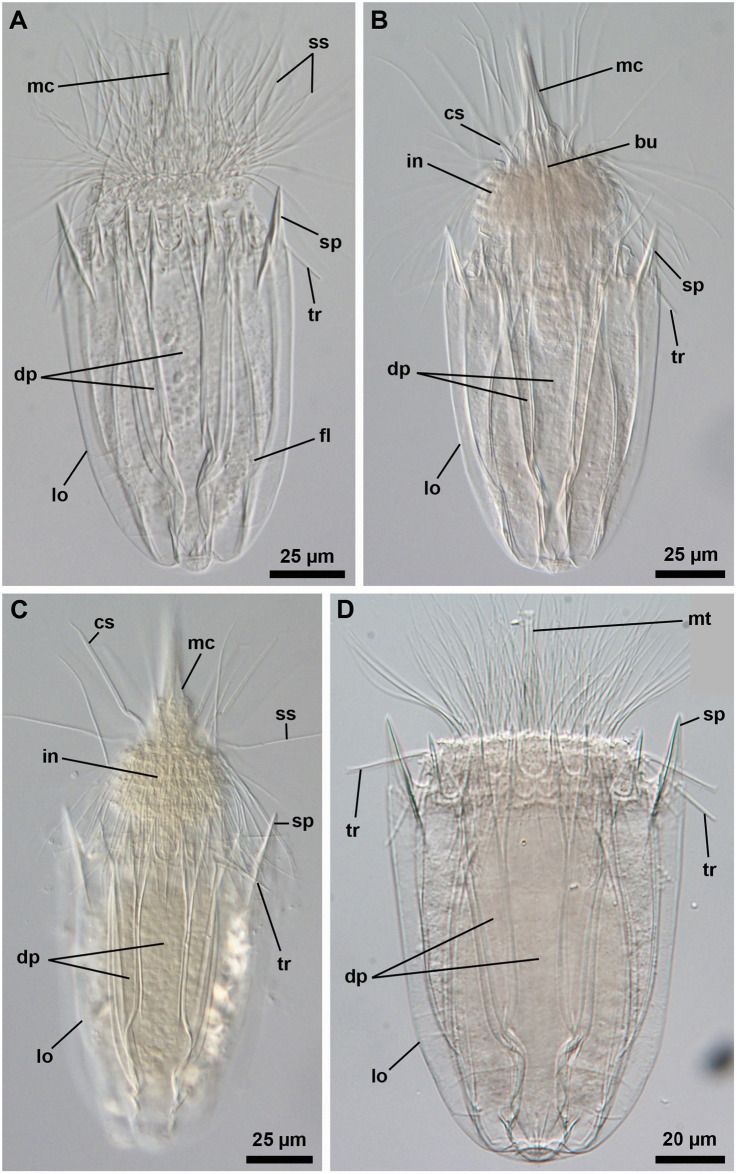
Light micrographs of four paratypic postlarvae belonging to genus *Nanaloricus*. Anterior faces up in all aspects. All specimens characterized by a dorsal plate divided into three subplates. (A and B) Postlarvae tentatively assigned to *Nanaloricus valdemari* sp. nov. (C and D) Postlarvae tentatively assigned to *Nanaloricus mathildeae* sp. nov. Abbreviations: bu, buccal tube; cs, clavoscalids; dp, dorsal plate; fl, flosculi; in, introvert; lo, lorica; mc, mouth cone; mt, mouth tube; sp, anterior spike; ss spinoscalid; tr, trichoscalid.

Besides the adult specimens used to describe the three new species reported here, additional postlarval specimens of Loricifera were also collected from the investigated area of Trezen ar Skoden (Fig 20; see above the list of paratypes of *Nanaloricus valdemari* sp. nov. and *Nanaloricus mathildeae* sp. nov. in which tentatively assigned postlarvae are included). Postlarval specimens are differentiated from adult forms by the dorsal plate, which is divided into three subplates through sclerotized longitudinal ridges that span the entire length of the lorica. In addition, only eight rows of scalids are present on the introvert, as the seventh row is absent. The postlarvae are also characterized by uniform clavoscalids that resemble those of the female, though no mature reproductive organs are present within the abdomen. Although all the sampled postlarvae clearly belong to family Nanaloricidae, these specimens could only be tentatively assigned to one of the new *Nanaloricus* species described herein, due to the lack of definite anatomical characters. However, the external morphology of some postlarval specimens (e.g., the shape of the longitudinal lines splitting the dorsal plate) seem similar to *N*. *valdemari* sp. nov. (Fig 20A and 20B), whereas others resemble *N*. *mathildeae* sp. nov. (Fig 20C and 20D) and the specimens have thus tentatively been assigned to species. None of the postlarval specimens found thus far seem to belong to *Scutiloricus hugoi* gen. et sp. nov.

## Discussion

### Differential diagnosis for *Nanaloricus valdemari* sp. nov. and *Nanaloricus mathildeae* sp. nov.

*Nanaloricus valdemari* sp. nov. and *Nanaloricus mathildeae* sp. nov. are distinguished from all other congeners, as well as between them, by the unique combination of a number of specific characters present in each of these two new species (see Table 1). Although the two new species clearly belong to *Nanaloricus*, distinctive features present in each of them are conspicuously different from all other species described so far in this genus, namely *Nanaloricus mysticus*, *Nanaloricus khaitatus* and *Nanaloricus gwenae* [8, 29, 30]. Noteworthy, a comprehensive comparison between the two new *Nanaloricus* species described here and *N*. *khaitatus* will not be provided at this time as the original description of the latter species ([29]; see also [31]) is most probably based on observations of specimens that belong to different species and perhaps even different genera (RMK, pers. obs.). Indeed, after all the knowledge acquired for Loricifera in the last decades, differences between the type specimens of *N*. *khaitatus* (e.g., the overall shape of the lorica) leave room for questioning the monospecificity of *N*. *khaitatus* (compare specimens shown on Fig 1 in [29]). Moreover, the holotype and the allotypic paratype of *N*. *khaitatus* are both mounted on SEM stubs, which would hamper a thorough comparison between them and specimens of *Nanaloricus valdemari* sp. nov. and *Nanaloricus mathildeae* sp. nov (e.g., only one side of the animal can be investigated). A more assertive taxonomical revision of *N*. *khaitatus* is necessary in the future, after a re-investigation of the type material as well as the collection of new material from the type locality. Indeed, sediments from the type locality of *N*. *khaitatus*–a 7 m deep-water site located in the Meloria shoals, off Livorno (Italy)–may well be much richer in loriciferan diversity than initially recognized.

**Table 1:** Comparison of adult characters of the species assigned to the genus *Nanaloricus* (family Nanaloricidae). *Nanaloricus khaitatus* is not included in this table because its original description is probably based on observations of at least three different species.

		*N*. *mysticus* [8, 15]	*N*. *gwenae* [30]	*N*. *valdemari* nov. sp. (this study)	*N*. *mathildeae* nov. sp. (this study)
**Mouth cone**:	**Length**	short	long	long	long
	**Honeycomb sculpture**	absent	absent	present	absent
	**Oral ridges**	8, of equal length	8, four primary oral ridges twice as long as the four secondary	8, four primary oral ridges one-fourth as long as four secondary	8, four primary oral ridges one-fourth as long as four secondary
**Introvert**:	**Female clavoscalids**	slender, with numerous papillae at the base and a filiform tip	slender, two-segmented, with several small, spike-like papillae at the base and a hook-shaped tip	slender, club-shaped, four-segmented, with many small papillae and a spinose tip	slender, club-shaped, four-segmented, with several long papillae and a spinose tip
	**Male clavoscalids**	multiform: midventral pair slender; six clavoscalids ramified into three branches; the tertiary branches broad and club-shaped (except in the laterodorsal pair)	? (males were never found)	multiform: midventral pair four-segmented, slender, with spinose tip; six clavoscalids ramified into three branches; the primary branch three-segmented, with spinose tip	multiform: midventral pair four-segmented, slender, with spinose tip; six clavoscalids ramified into three broad, flat branches; the primary branch three-segmented, with spinose tip
	**8**^**th**^ **row spinoscalids**	unsegmented, whip-like	two-segmented, distal segment with serrated margins	unsegmented, whip-like	unsegmented, whip-like
**Neck**:	**Double and single trichoscalids**	all with blunt tips, uniform; in males, the upper row appendages of the lateroventral pair is modified into claspers (claw-shaped organs)	all with blunt tips, but upper row appendages of the double trichoscalids are slightly thinner than lower row	all with blunt tips, but upper row appendages of the double trichoscalids are longer (ventrolateral pairs) or slightly longer (lateral pairs) than lower row	upper row appendages of the double trichoscalids have a pointy tip and are longer than those in the lower row, which have a blunt tip; single trichoscalids are broader than the double trichocalids, and possess a unique blunt tip with several (ca. 14?) finger-like processes
	**Sensory organ of the double trichoscalids**	not found	not found (though a short, spine-like structure is present in the drawings of this species description)	present, located at the most anterior margin of the upper appendage basal plate of the ventral and ventrolateral double trichoscalids	present, located anteroproximally to the ventral and ventrolateral double trichoscalids, and protrudes from its own plate with a double-square shape
**Lorica**:	**Nr. of plates**	6	6	6	6
	**Anterior spikes**	15 (midventral spike short)	15 (midventral spike short)	15 (midventral spike very short)	15 (midventral spike very short)
	**Honeycomb sculpture**	present	present	present	present
	**Number of N-flosculi**	4 large, arranged rectangularly on each dorsolateral plate; 1 large, located posteriorly on the dorsal plate	4 large, arranged rectangularly on each dorsolateral plate; 1 large, located posteriorly on the dorsal plate	4 large, arranged rectangularly on each dorsolateral plate; 1 large, located posteriorly on the dorsal plate	4 large, arranged rectangularly on each dorsolateral plate; 1 large, located posteriorly on the dorsal plate

The presence of a pair of longitudinal stripes spanning along the anterior two thirds of the dorsal plate is a feature found exclusively in *N*. *valdemari* sp. nov. This anatomical feature has not previously been observed in any other adult loriciferan. Another unique feature observed in this species is the well-defined honeycomb sculpture adorning the mouth tube. This condition was never observed before, neither in adult forms nor in larval stages belonging to Nanaloricidae, in which the honeycomb sculpture is exclusive of the lorica. As for the gross morphology of *Nanaloricus mathildeae* sp. nov., the presence of very broad clavoscalids in the male form is a unique species-specific character. Species of Nanaloricidae are known to possess sexually dimorphic clavoscalids and the first described loriciferan species, *N*. *mysticus*, is characterized by males with relatively broad clavoscalids (secondary branch is ca. 4.5 μm wide). However, the clavoscalids found in the species described here are broader (secondary branch is ca. 8 μm wide) than those of *N*. *mysticus*, a feature that is easily confirmed by using either light or scanning electron microscopy. Another diagnostic trait present in *Nanaloricus mathildeae* sp. nov. is the presence of a cuticularized bar preceding each of the oral furcae in adult forms, a condition never observed before in Nanaloricidae.

A feature present in both *Nanaloricus* species described here that attracts attention is the presence of a sensory organ associated with the double trichoscalids located ventrally and ventrolaterally. Although anatomically identical, the double trichoscalid sensory organ of *N*. *valdemari* sp. nov. is located at the most anterior margin of the upper trichoscalid plate, while in *N*. *mathildeae* sp. nov. it protrudes from its own cuticular plate. The relative position of the double trichoscalid sensory organ, as well as the presence/absence of a related basal plate, is thus source for important differences between the two *Nanaloricus* species described here. This sensory structure is also morphologically similar to the so-called claspers or claw-shaped organs found in males of *Nanaloricus mysticus* [15]. However, the latter organs have a short peduncle and replace the upper appendage of the lateroventral double trichoscalids, which is in striking contrast with the condition found in the two new *Nanaloricus* species described here. These claspers, as the name indicates, are thought to have a functional role during copulation. Another interesting feature found in both *N*. *valdemari* sp. nov. and *N*. *mathildeae* sp. nov. is the telescopic mouth tube. This feature was known from only a couple species of Nanaloricidae, namely *Armorloricus elegans* [28] and *Spinoloricus cinziae* (see Figs 9–11 in [18]), but the discovery of two more species (and a third genus) with this ability raises the hypothesis that all species of Nanaloricidae possess a telescopic mouth tube. However, new observations on all previously described species are needed in order to confirm this hypothesis.

Overall, the external morphology of *N*. *valdemari* sp. nov. and *N*. *mathildeae* sp. nov. reveals a high degree of resemblance and, in turn, both species are more similar to *Nanaloricus mysticus* than to *Nanaloricus gwenae* [8, 30]. However, it should be stressed that males of *N*. *gwenae* were never found, which hampers a more comprehensive comparative analysis between all four species. The latter further indicates that males are rarer than females in *Nanaloricus*–an observation, which is substanciated by the current species descriptions, in which females are clearly overrepresented.

### Differential diagnosis of *Scutiloricus hugoi* gen. et sp. nov.

*Scutiloricus hugoi* gen. et sp. nov. can be distinguished from all other genera assigned to Nanaloricidae—namely *Nanaloricus*, *Armorloricus*, *Phoeniciloricus*, *Spinoloricus*, *Culexiregiloricus* and the recently described *Fafnirloricus* [8, 13, 28, 32–34]–by its possession of a unique combination of characters as outlined in Table 2. This table, moreover, provides further references to formal descriptions of additional species assigned to the various genera; [18, 19, 30, 35]. *Australoricus*, a genus that was discovered in sea caves off New South Wales in Australia, is not included because its description is based solely on the larval stage [36]. Indeed, *Scutiloricus hugoi* gen. et sp. nov. can be distinguished from all other loriciferans by its possession of a combination of distinctive morphological features that includes: (i) fenestrated anterior spikes, (ii) linear arrangement of the dorsolateral flosculi, (iii) slightly invaginated anal field with a small anal cone flanked by a pair of small spurs, and (iv) females with a unique pair of seminal receptacles.

**Table 2:** Comparison of adult (or in certain cases postlarval) morphological characters between the genera assigned to family Nanaloricidae.

		*Nanaloricus* [8, 30]*	*Armorloricus* [28, 35]	*Phoeniciloricus* [32]	*Spinoloricus* [18, 19, 33]	*Culexiregiloricus* [34]	*Fafnirloricus* [13]	*Scutiloricus* gen. nov. (this study)
**Mouth cone**:	**Length**	short	very long	long	short	long	very long	long
	**Oral ridges**	short and long (two types)	all short	all short	all short	all short	all short	short and long (two types)
	**Oral stylets**	absent	present	present	absent	present	absent	absent
	**Pleat**	absent	absent	absent	present (but absent in *S*. *cinziae*^a^)	absent	absent	absent
**Introvert**:	**9**^**th**^ **row of spinoscalids**	teeth-like	beak-like	beak-like	beak-like	beak-like	beak-like	teeth-like
	**Alternating plates**	absent	absent	absent	present (with 9^th^ row of spinoscalids)	absent	present (with 8^th^ row of spinoscalids)	absent
**Lorica**:	**Overall shape**	oval	square	oval	oval	oval	oval	square
	**Honeycomb sculpture**	present	absent	present	present	present	absent	present
	**Nr. of plates**	6	6	10	6	8	20	6
	**Anterior spikes (length)**	15 (all long, except the midventral one)	15 (long)	15 (short)	14 (short) + 12 additional spikes	14 (short)	14 (short)	14 (long)
	**Intercalary plicae**	absent	absent	absent	present	present	absent	absent
	**Number of N-flosculi**	9 or 10; 4 large on each dorsolateral plate and 1 large or 2 microflosculi on the dorsal plate	9; 4 large on each dorsolateral plate and 1 microflosculi on the dorsal plate	18 (all large); 4 on each dorsolateral plate and 2 on the dorsal plate; 4 additional pairs located latero-ventrally	7; 3 large on each dorsolateral plate and 1 small on the dorsal plate	8 (all large); 3 on each dorsolateral plate and 2 on the dorsal plate	12 (all large); 4 anteriorly and 2 posteriorly on each dorsolateral plate	10 (all large); 4 on each dorsolateral plate and 2 on the dorsal plate
	**Arrangement of dorsolateral N-flosculi**	rectangular	rectangular	rectangular	triangular	triangular	rectangular^b^	linear
	**Furrow amid flosculi**	absent	absent	absent	present	present	absent	absent
	**Anal field**	small, triangular anal plate located dorsally; anal cone is very small or indistinct	blunt or round posteriormost region of the dorsal plate; anal cone composed of 3 anal plates that cover anus	composed of a double anal plate, which protrudes and forms a shield, covering the anus dorsally	composed of 6 small plates; with a slightly protruding anal cone	ventral anal region with a small but well-defined, protruding, pointy anal cone	posterior, middorsal depression with a relatively wide anal cone	slightly invaginated, with narrow anal cone protruding posteroventrally and flanked by a pair of small spurs
**Internal structures**:	**Seminal receptacles**	not found	not found	not found	not found	not found	not found	present

Characters of *Phoeniciloricus* and *Culexiregiloricus* are based on descriptions of the postlarval stage as adult stages are not known for these two genera. The nanaloricid genus *Australoricus* is not included in this table because neither the postlarval nor the adult stages are known for this genus (i.e., its description is based solely on the larval stage).

*Characters from *Nanaloricus khaitatus* were not taken into account in this comparative table because the original description of the species is probably based on observations of at least three different species.

^a^ See [18].

^b^ Concerning only the anteriormost groups of flosculi.

Two other morphological features attract special attention in *Scutiloricus hugoi* gen. et sp. nov., namely the arrangement of the dorsolateral flosculi and the peculiar anatomy of its invaginated anal field. The arrangement of dorsolateral flosculi has traditionally been used as a diagnostic character for comparative morphology of Loricifera. Among all genera assigned to Nanaloricidae, *Scutiloricus hugoi* gen. et sp. nov. is the only genus possessing linearly arranged dorsolateral flosculi. Specifically, all Nanaloricidae genera described so far possess either a rectangular or a triangular arrangement of dorsolateral flosculi. The linear pattern found in *Scutiloricus hugoi* gen. et sp. nov. is thus a unique feature described for the first time in Loricifera. The anal field with a small, protruding anal cone flanked by a pair of spurs that characterizes this new genus also represents a very peculiar combination of traits. Although a similar anal cone can be found in *Culexiregiloricus* [34], no other genus belonging to Nanaloricidae is characterized by an invaginated anal field with a pair of spurs, as observed in *Scutiloricus hugoi* gen. et sp. nov. In addition, there are almost no other similarities with *Culexiregiloricus*, a genus that is characterized by an oval lorica with intercalary plicae and dorsolateral flosculi arranged triangularly with a furrow located amid them. Overall, the external morphology of the new genus and species described here is not similar to any of the other generaassigned to Nanaloricidae (for a comparative analysis see Table 2). Indeed, only isolated features from each of the body sections are shared between *Scutiloricus hugoi* gen. et sp. nov. and other nanaloricid genera. For instance, both the new genus and species described here and *Armorloricus* possess a square lorica [28]; however, the latter genus has 15 anterior spikes (ventral plate has three), whereas *Scutiloricus hugoi* gen. et sp. nov. possesses only 14 (ventral plate has two).

Besides the external features, an aspect of the internal anatomy of *Scutiloricus hugoi* gen. et sp. nov. requires special attention. The finding of seminal receptacles in a female of this new genus and species is a first time observation for Loricifera, providing evidence for the existence of a female sperm-storage organ in this group of microscopic invertebrates. Moreover, the presence of mature sperm inside a paratypic female demonstrates that the seminal receptacles were functional at the time the specimen was preserved. Interestingly, though, ultrastructural observations of *Armorloricus elegans* provided evidence for the presence of spermatozoa inside a female specimen [37]. However, the presence of seminal receptacles in this species was never assessed. In the future, the study of this female organ is necessary to understand the functional morphology and ultrastructure of the newly described seminal receptacles, as well as to better comprehend the reproductive activity and sexual selection in Loricifera.

### Final remarks

Loriciferans are strictly marine organisms inhabiting sediments across the globe from tidal to deep hadal zones [e.g. 16]. The phylum is represented by a single order, Nanaloricida, and currently comprises three families, Nanaloricidae, Pliciloricidae and Urnaloricidae, harboring 42 described species (including the three new nanaloricid species described herein) [8–14]. In addition, the so-called Shira-larva, assigned to its own species of uncertain placement, currently resides outside the three loriciferan families [25].

The marine sediments off the coast of Roscoff (France) are well known for the presence of Loricifera. Importantly, specimens of the first described loriciferan species, *Nanaloricus mysticus*, were found in this area at 25–30 m water depths in a sediment type known as “*Dentalium* sand” (48°43’N-03°54’W; [8]). Two other species belonging to a different nanaloricid genus, *Armorloricus elegans* and *Armorloricus davidi*, were subsequently discovered from the large shell dune known as Trezen ar Skoden (48°45’55’’N- 04°06’45’’W; [28]), located also in the coastal area off Roscoff [38]. The specimens investigated for the description of these two species were collected from fine shell gravel (with *Polygordius*). Besides these nanaloricids, a single larval specimen belonging to another family, Urnaloricidae, was also found in the vicinity of Roscoff, more specifically in the Bay of Morlaix (RMK, pers. obs.). This larva was found in maerl (*Lithothamnion*) sand collected in 2004. With the three new species and new genus described in the present study, the number of loriciferan species present in the coastal area off Roscoff increases to seven. To our knowledge this subtidal area is thus among the most biodiverse localities in the world regarding loriciferan fauna. Interestringly, this observation also holds for other meiofauna groups, as the Roscoff area also represents a biodiversity hotspot for e.g. tardigrades [5, 7]. Other loriciferan biodiversity hotspots include the sandy habitats (289–439 m water depths) off the coast of North and South Carolina (USA) from where Pliciloricidae was originally described [9] as well as the Faroe Bank, located south of Faroe Islands, from where Urnaloricidae was erected based on a species found in clean carbonated white sand at 120–260 m water depths [10]. Fascinatingly, urnaloricids seem to lack traditional adult female and male stages, and Higgins larvae apparently develop from oocytes formed within a so-called mega-larva. Most intriguingly, loriciferans have also been found in various extreme marine environments including the hypersaline anoxic deep basin of the Mediterranean Sea [18, 39] as well as in connection with deep sea manganese nodules [13].

The description of the three new loriciferan species reported here stems from several decades of sampling in the coastal area off Roscoff. Specifically, the collection of specimens used in the description of these new species spanned 35 years, i.e. the period of 1985–2020. Although the exact number of specimens of *N*. *valdemari* sp. nov. and *N*. *mathildeae* sp. nov. used in previous molecular and morphological studies [26, 27] is not known with precision, several dozen animals—including different life cycle stages—were collected and used in both morphological and molecular studies. This is in clear contrast with the few specimens of *Scutiloricus hugoi* gen. et sp. nov. found thus far. Only four specimens belonging to this new species and genus have been found until now: two females (paratypes) in July 1985, a male (allotypic paratype) in December 2013 and a female (holotype) in May 2013. The reasons behind this apparently low population density are yet to be unraveled, though more sampling efforts not only in Trezen ar Skoden, but also in the surrounding area, could provide new insights into the distribution of the new species and genus in the large coastal area off Roscoff.

In addition to adult forms and postlarval stages, Higgins larva specimens clearly belonging to four different species were found in sediment collected from Trezen ar Skoden between 1985 and 2020. Unfortunately, the larval specimens were often found in samples containing adults of more than one nanaloricid species. Two of the four larval morphotypes were initially described as *Armorloricus* sp. I and *Armorloricus* sp. II [28]. These species were later assigned to *Armorloricus elegans* and *Armorloricus davidi*, respectively, based on the consistent and recurrent finding of larval morphotypes together with adult forms of only one species [12]. The other two larval morphotypes clearly belong to genus *Nanaloricus* and are, to some extent, similar to the Higgins larva of *Nanaloricus mysticus*. These larval morphotypes are always found in samples holding *N*. *valdemari* sp. nov. and *N*. *mathildeae* sp. nov., though an assignment to either species has not yet been possible.

Finally, the description of *Scutiloricus hugoi* gen. et sp. nov. is relevant for the diversity of Loricifera. As compared with Pliciloricidae (with three genera) and Urnaloricidae (with one genus), Nanaloricidae is now the most diverse of the three loriciferan families, accommodating a total number of seven genera. Recently described, the new species and genus *Fafnirloricus polymetallicus* provided new insights into how diverse the nanaloricid anatomy can be [13]. For instance, new oral structures were described for the first time and the highest number of lorica plates, i.e. 20, was reported for a member of Nanaloricidae. In addition, an atypical arrangement of the introvert scalids was suggested for *F*. *polymetallicus*. In this regard, *Scutiloricus hugoi* gen. et sp. nov. possesses a more traditional nanaloricid-like anatomy. However, its unique anal field and female seminal receptacles add important novelties to our knowledge of the nanaloricid body plan.

## Supporting information

S1 Video Live adult female of *Nanaloricus mathildeae* sp. nov. in motion. Same specimen as shown in Fig 13; dorsal view. Note the different stages of body retractions, while the animal moves. Internally, note the pharyngeal bulb and the presence of two ovaries containing oocytes.
(MP4)
